# Disadvantages of the Use of Low-Protein Diets in Weaned Piglets and Nutritional Interventions: A Meta-Analysis

**DOI:** 10.3390/ani16081157

**Published:** 2026-04-10

**Authors:** Jingchun Gao, Xiaoyi Long, Qingsong Tang, Xie Peng, Yetong Xu, Zhihong Sun

**Affiliations:** 1 Research Center for Bio-Feed and Molecular Nutrition, College of Animal Science and Technology, Southwest University, Chongqing 400715, China; gaojingchun2021@126.com (J.G.); longxy8350@163.com (X.L.); tqs5213@foxmail.com (Q.T.); pengxie2022@swu.edu.cn (X.P.); xyt8501@163.com (Y.X.); 2 Key Laboratory of Chongqing Education Animal Nutrition and Bio-Feed, Chongqing 400715, China

**Keywords:** growth performance, intestinal morphology, low-protein diet, meta-analysis, weaned piglets

## Abstract

This study aimed to systematically evaluate the effects of low-protein (LP) diets and their associated nutritional interventions on growth performance and intestinal health in weaned piglets through a meta-analysis. Following the PRISMA guidelines, 21 studies were selected from an initial set of 1720 to construct a database. A random-effects model was employed to assess the growth performance and intestinal health of weaned piglets, comparing LP diet with control diet, and LP diet + additive with LP diet alone. Heterogeneity was assessed using the I^2^ statistic, and its sources along with publication bias were explored through meta-regression, subgroup analysis, and Egger’s linear regression. The results show that LP diets (crude protein < 18%) reduced final body weight (*p* < 0.001), average daily gain (*p* < 0.001), and gain-to-feed ratio (*p* < 0.001) of weaned piglets. Additionally, forest plot data show that LP diet (crude protein < 18%) decreased villus height and crypt depth in the jejunum and ileum of weaned piglets (*p* < 0.05). To mitigate these adverse effects, plant extracts, amino acids (AAs), fatty acids, vitamins, enzymes, and carbohydrates are commonly used as additives. Among these, the results show that LP diets supplemented with AAs and plant extracts increased average daily feed intake (*p* < 0.001) and ileal crypt depth (*p* < 0.05). Network meta-analysis further identified AAs and plant extracts as the most effective additives for improving growth performance of weaned piglets. In conclusion, dietary protein levels below 18% negatively affect the growth performance of weaned piglets, and supplementation with AAs or plant extracts represents a promising strategy to counteract these adverse effects.

## Simple Summary

This systematic review and meta-analysis (21 studies, PRISMA protocol) examines the effects of low-protein diets in weaned piglets and evaluated nutritional countermeasures. Low-protein diets reduced growth performance, including average daily gain, feed efficiency and final weight, and compromised intestinal morphology, as evidenced by reduced jejunal and ileal villus height and crypt depth. Meta-analytic results show that adding amino acids or plant extracts to low-protein diets improved feed intake and increased ileal crypt depth. The review further identifies three principal nutritional strategies to offset the drawbacks of low-protein feeding: adopting net energy formulation, improving amino acid utilization, and optimizing feed processing and formulation.

## 1. Introduction

The livestock and poultry industry faces challenges due to protein feed shortages, inefficient nitrogen utilization, and environmental pollution problems [1]. The application of low-protein (LP) diets positively contributes to reducing feed ingredient costs and alleviating environmental pollution in weaned piglets. LP diets are defined as diets in which the crude protein (CP) level is reduced while appropriate types and amounts of industrial amino acids (AAs) are supplemented, without compromising growth performance or product quality. Currently, there is still no established definition specifying a particular CP level for LP diets, as the nutritional standards followed may vary by region. Generally, in the scientific literature, a diet with a CP level reduced by more than 1.5% compared to a normal-protein diet are considered LP diets. However, excessive reduction in dietary CP levels has been shown to result in growth retardation and increased fat deposition [2,3,4].

In the past few decades, many studies have explored the effects of reducing dietary protein on the growth performance and intestinal morphology of weaned piglets [5]. Studies have shown that reducing dietary CP levels from 20% to 16% results in a decreasing trend in both the diarrhea incidence and diarrhea index of weaned piglets [3]. Studies have reported that reducing dietary CP levels from 23% to either 19% or 17% results in decreased average daily gain (ADG) and average daily feed intake (ADFI) in weaned piglets [6,7]. Furthermore, studies have found that reducing dietary CP levels from 20% to 14% further decreases small intestinal villus height and the ratio of villus height to crypt depth, leading to intestinal morphological damage [8]. To address the contradictory findings in the existing literature regarding the effects of LP diets on growth performance and intestinal morphology in pigs, many researchers have conducted further investigations and reviews, aiming to provide references for practical formulation strategies in the swine industry [4,9]. Although several meta-analyses have been conducted on LP diets and AA supplementation, these studies have primarily focused on the effects of reducing CP levels and supplementing crystalline AAs in growing–finishing pigs. To date, there remains a lack of meta-analyses specifically examining the effects of LP diets on growth performance and intestinal health in weaned piglets. Additionally, given the potential negative effects associated with the use of LP diets in weaned piglets, summarizing the regulatory roles of various feed additives in piglets fed LP diets holds an important reference value for future research.

In piglet rearing, feed additives such as plant extracts, fatty acids, and AAs are commonly used to enhance nutrient utilization, which in turn reduces nitrogen excretion [10,11]. The use of supplemental AAs helps to achieve the ideal AA profile and further reduces CP levels in the diet, improving nitrogen utilization and pig growth [12,13]. Studies have shown that LP diets supplemented with feed additives improve the growth performance and intestinal morphology of weaned piglets and enhanced immunity [2,14,15,16]. A study also found that feeding LP diets supplemented with sodium butyrate improved the immunity and antioxidant capacity of weaned piglets [14]. However, the effects of different categories of feed additives under varying levels of CP reduction on weaned piglets have not yet been systematically evaluated. Meta-analysis is a statistical method that synthesizes results from multiple experiments to estimate treatment effects more accurately and investigate potential heterogeneity between experiments, thereby enhancing the generalizability of conclusions [17,18,19]. This study aimed to use meta-analysis to investigate the effects of LP diets on the growth performance and intestinal health of weaned piglets and to identify effective feed additives that mitigate the negative impact associated with feeding LP diets to weaned piglets. This meta-analysis enabled an explicit investigation of the interaction between the magnitude of CP reduction and the inclusion of different additive types as potential sources of heterogeneity. Additionally, meta-regression and subgroup analyses were employed to identify key factors influencing the response to LP diets, including animal age, experimental duration, dietary CP levels, and the magnitude of protein reduction.

## 2. Materials and Methods

### 2.1. Literature Search Strategy, Inclusion Criteria, and Screening Process

**Figure 1:**
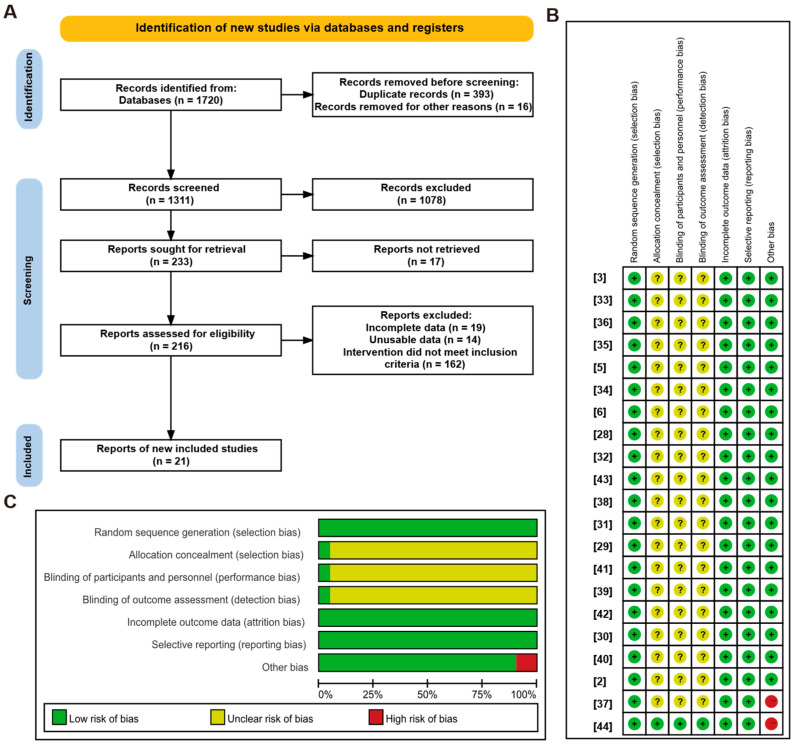
Flow of the literature search, screening, and study quality assessment. (**A**) Flow chart of the literature search and screening; (**B**) risk of bias graph; (**C**) risk of bias summary. + (green) = low risk of bias; - (red) = high risk of bias; ? (yellow) = unclear risk of bias.

This meta-analysis was conducted in accordance with the Preferred Reporting Items for Systematic Reviews and Meta-Analyses (PRISMA) 2020 statement [20,21]. The review was not registered. The completed PRISMA 2020 checklist is provided in the Supplementary Materials. A comprehensive search of the PubMed, Web of Science, and Science Direct databases was conducted for articles published between 1 January 2000 and 1 January 2024 to identify studies employing LP diets in weaned piglets. The complete search strategies are presented in Table S1.1.1. Additionally, Figure 1A provides a detailed overview of the literature screening process.

This meta-analysis included studies that met the following criteria: (1) The growth performance and intestinal health parameters were reported, including the ADG, average daily feed intake (ADFI), feed conversion ratio (feed-to-gain ratio), gain-to-feed ratio, villus height (VH), crypt depth (CD) and villus height: crypt depth ratio (V:C ratio). (2) The experiments were conducted on weaned piglets. (3) The gap in CP content between the low-CP and normal-CP diets exceeded 1.5%, and the normal-CP diet was formulated in accordance with NRC 1998, NRC 2012, NSNG V2.0, CVB 2003, and the Danish Nutrient Requirement Standards. The following were the exclusion criteria: (1) the study lacked corresponding data, (2) the experiments had added antibiotic growth promoters, and (3) the initial weight was greater than 9 kg. We independently verified the CP contents of both low CP and normal CP diets reported in each included study and cross-checked these against the nutritional requirement standards referenced in the original studies. This verification was performed independently by two investigators (J. Gao and X. Long), and any discrepancies were resolved through discussion to reach a consensus. We strictly followed the above criteria and screened eligible studies for inclusion in the analysis. Information extracted from the included experiments was as follows: author information (first author, year); sample size of pigs included in each of the control and treatment groups; weaning age; CP levels in each of the control and treatment groups; mean initial body weight; mean final body weight; experiment duration; supplemental substance and concentration; growth performance (ADG, ADFI, feed-to-gain ratio, and gain-to-feed ratio), fecal consistency, intestinal morphology (VH, CD, V:C ratio).

### 2.2. Data Analysis

#### 2.2.1. Study Quality Assessment

Figure S1 shows the distribution of sampling study areas around the world (Methods in the Supplementary Materials). Two investigators (J. Gao and X. Long) conducted an independent study quality assessment and data extraction. The assessment encompassed the following aspects: sequence generation, allocation concealment, blinding of participants and personnel, incomplete outcome data, selective outcome reporting, and other biases. Any differences of opinion were resolved by reaching a consensus.

#### 2.2.2. Heterogeneity and Effect Size

Meta-analysis requires within-group standard deviations (SDs) or standard errors (SEs), which were often missing in the included studies. For studies reporting the mean difference (MD) and *p* value but not the SD, the pooled SD was estimated assuming equal variances between groups. The t value was derived from the two-tailed *p* value and degrees of freedom (df = N_E_ + N_C_ − 2) using the inverse t distribution. For *p* < 0.05 or *p* < 0.01, the upper bound (0.05 or 0.01) was used. The standard error of the MD was calculated as

$$SE=\frac{\left|MD\right|}{t}$$

and the pooled SD as

$$SD=\frac{SE}{\sqrt{\frac{1}{N_{E}}+\frac{1}{N_{C}}}}$$

This approach follows the *Cochrane Handbook for Systematic Reviews of Interventions* [22,23]. We used a random-effects model to calculate the pooled estimate of the standard mean difference (SMD) with the 95% confidence interval (CI). If the 95% CI included a value of zero, this result indicated that there was no difference. Heterogeneity was assessed using the I^2^ statistic and Cochran’s Q test; I^2^ > 50% was considered substantial heterogeneity. Subgroup analyses were then performed to explore potential sources of heterogeneity [24,25,26]. Publication bias was assessed using Egger’s tests, with a significance level of *p* < 0.1 [18]. Publication bias was assessed graphically using funnel plots [27].

#### 2.2.3. Sensitivity Analysis and Surface Under the Cumulative Ranking Curve

We conducted a leave-one-out sensitivity analysis by iteratively omitting each study and recalculating the pooled standard mean difference (SMD) with its 95% CI. This procedure evaluates the influence of individual studies on the overall effect estimate. All analyses used a random-effects model in STATA version 17 employing the “metan” command, with the “metaninf” routine applied for the leave-one-out diagnostics.

The Markov chain Monte Carlo method was employed to derive the most accurate combined estimates and probabilities for each intervention. The surface under the cumulative ranking curve (SUCRA) was also calculated for cumulative probability ranking. In this context, the closer SUCRA was to 1, the more pronounced the beneficial effect of the additive intervention, whereas the closer it was to 0, the more detrimental the effect.

Analysis was performed using R version 4.2.2 and Stata 17.0; detailed data information is shown in the Supplementary Materials.

## 3. Results

A meta-analysis was used to evaluate the effects of LP diets on the growth performance and intestinal morphology of weaned piglets over the past few decades. We identified 1720 articles in Science Direct (191), Web of Science (1225), and PubMed (304), of which 21 were included in the meta-analysis [2,3,5,6,28,29,30,31,32,33,34,35,36,37,38,39,40,41,42,43,44]. Figure 1A illustrates the filtering steps and reasons for exclusion, and Tables S1.1.2 and S1.1.3 summarize the characteristics and outcomes of the studies. Additionally, the risk of bias assessment for the included studies is shown in Figure 1B,C. Among the included studies, 19 experiments showed unclear risk of bias, and 2 showed a high risk of bias (Table S2.1.1). Furthermore, the effect of reducing dietary protein levels on the growth performance of weaned piglets was determined by comparing the LP diets with the corresponding control group.

### 3.1. Effects of LP Diets on the Growth Performance and Intestinal Morphology of Weaned Piglets

#### 3.1.1. Growth Performance

**Figure 2:**
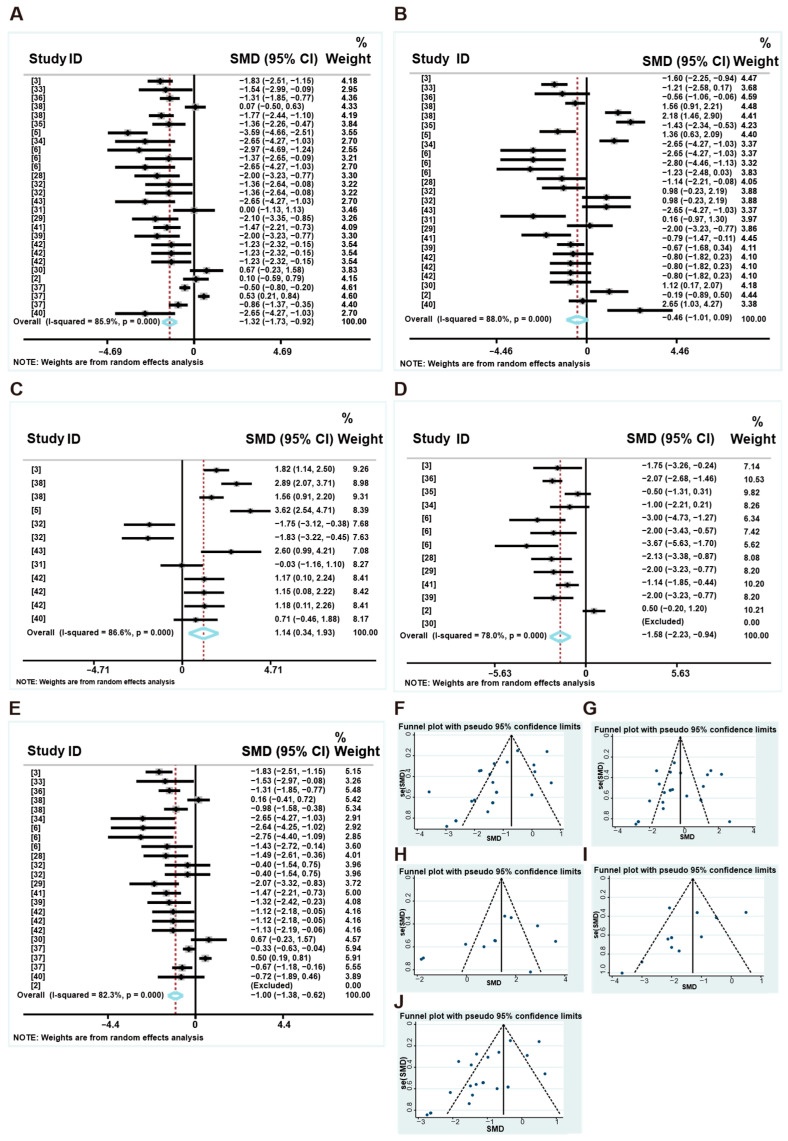
Forest plot of the effects of LP diets on the growth performance of weaned piglets. Forest plot of the effects of LP diets on ADG (**A**), ADFI (**B**), feed-to-gain ratio (**C**), gain-to-feed ratio (**D**), and final BW (**E**) in weaned piglets. Funnel plot of the effects of LP diets on ADG (**F**), ADFI (**G**), feed-to-gain ratio (**H**), gain-to-feed ratio (**I**), and final BW (**J**) in weaned piglets. Control group = normal protein diet; experimental group = LP diet; SMD = standard mean difference; 95%CI = 95% confidence interval; I^2^ (0~100%) = test for heterogeneity. Test for overall effect: *p* value (significance level: *p* < 0.05). Diamonds on the positive quadrant of the X-axis indicate an increase in the growth parameters, whereas those on the negative quadrant indicate a decrease.

Figure 2 shows the overall effects of LP diets on the growth performance of weaned piglets. Specifically, the forest plot reveals that LP diets reduced the ADG of weaned piglets (standard mean difference (SMD): −1.322, 95% CI: −1.728 to −0.916, *p* < 0.001) compared to the control group, with high heterogeneity (I^2^ = 85.9%) (Figure 2A). The funnel plot and Egger’s linear regression results indicate a significant publication bias (*p* < 0.001) (Figure 2F and Table S3.1.1). However, compared to the control group, it had no significant effect on the ADFI of weaned piglets (SMD: −0.457, 95%CI: (−1.007 to 0.093), *p* = 0.103), with high heterogeneity (I^2^ = 88%) and no significant publication bias (*p* = 0.180). (Figure 2B,G). LP diets increased the feed-to-gain ratio (SMD: 1.136, 95%CI: (0.338 to 1.935), *p* = 0.005), with high heterogeneity (I^2^ = 86.6%) (Figure 2C), and reduced the gain-to-feed ratio (SMD: −1.584, 95%CI: (−2.233 to −0.936), *p* < 0.001), with high heterogeneity (I^2^ = 78%) (Figure 2D). The funnel plots and Egger linear regression results indicate no significant publication bias (*p* > 0.05) (Figure 2H,I and Table S3.1.1). Furthermore, LP diets reduced the final body weight (final BW) of weaned piglets (SMD: −1.003, 95%CI: (−1.384 to −0.622), *p* < 0.001), with high heterogeneity (I^2^ = 82.3%) (Figure 2E). The funnel plot and Egger’s linear regression results indicate a significant publication bias (*p* = 0.0001) (Figure 2J and Table S3.1.1). Moreover, the leave-one-out sensitivity meta-analysis demonstrated that omitting any single study did not alter the nonsignificant finding, further supporting the robustness of the pooled estimates (Tables S3.2.1–S3.2.5). The trim-and-fill method revealed 9 missing observations for the ADG and 11 missing observations for the ADFI in the funnel plot (Figure S2).

#### 3.1.2. Subgroup Analysis for Growth Performance

**Figure 3:**
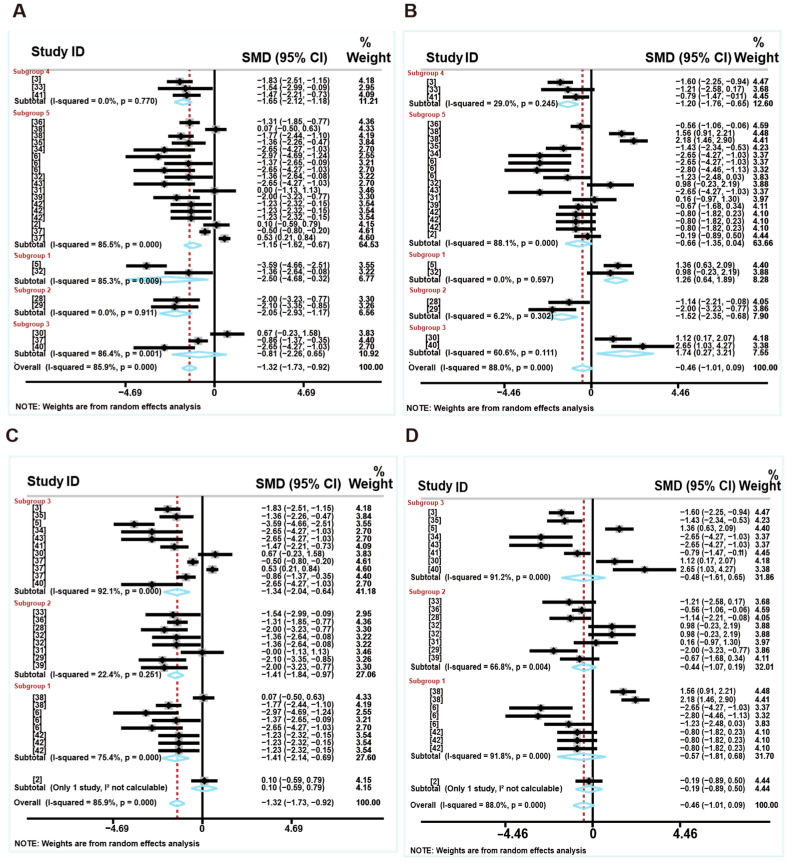
Subgroup analysis of different protein levels and weaning age forest plot of the effects of LP diets on growth performance in weaned piglets. Subgroup analysis of ADG (**A**) and ADFI (**B**) in weaned piglets (subgroup 1 is 13%CP; subgroup 2 is 14% CP; subgroup 3 is 15% CP; subgroup 4 is 16% CP; subgroup 5 is 17% CP). Subgroup analysis of ADG (**C**) and ADFI (**D**) in weaned piglets (subgroup 1 is “<21days”; subgroup 2 is “=21days”; subgroup 3 is “>21days”). Control group = normal protein diet; experimental group = LP diet; SMD = standard mean difference; 95%CI = 95% confidence interval; I^2^ (0~100%) = test for heterogeneity. Test for overall effect: *p* value (significance level: *p* < 0.05). Diamonds on the positive quadrant of the X-axis indicate an increase in the growth parameters, whereas those on the negative quadrant indicate a decrease.

These experiments included different levels of protein reduction to 13%, 14%, 15%, 16%, and 17%, different weaning ages (<21 days, =21 days, and >21 days), different treatment duration (<4 weeks and ≥4 weeks) and different initial BW (<8 kg and ≥8 kg). Experimental variation may influence heterogeneity and outcome; we divided the subgroups into different levels of dietary protein reduction, weaning age, treatment duration and initial BW (Figure 3, Figures S2 and Table S3.3.1). Sources of heterogeneity were further explored.

As shown in Figure 3A, Subgroups 1–5 respectively correspond to 13% CP-17% CP. Except for the 15% subgroup (*p* > 0.05), the different CP levels reduction reduced ADG compared to the control group, and the 17% subgroup had the lowest SMD values (*p* < 0.05). Meanwhile, as presented in Figure 3B, Subgroups 1–5 respectively correspond to 13% CP-17% CP. Compared to the control group, subgroups comprising 17% had no significant effect on the ADFI of weaned piglets (*p* > 0.05), while subgroups comprising 15% and 13% showed an increase in the ADFI of weaned piglets (*p* < 0.05). However, the subgroup comprising 14% showed a reduction in ADFI (*p* < 0.05) (Figure 3B). Figure 3C,D show the effects of different weaning ages on the growth performance of weaned piglets that were fed LP diets. We found that except for the <21 days subgroup, the different weaning ages reduced ADG compared to the control group (*p* < 0.05) (Figure 3C), and weaning ages were not the primary causes of heterogeneity. Meanwhile, as shown in Figure 3D, compared to the control group, the subgroups had no significant effect on the ADFI of weaned piglets (*p* > 0.05), and grouping factors were not a source of heterogeneity. Furthermore, we conducted meta-regression analyses to investigate the sources of heterogeneity further. The results of the study demonstrate that neither protein levels nor weaning age were responsible for the high heterogeneity observed in ADG and ADFI (*p* > 0.05). To evaluate the influence of treatment duration as a covariate on the pooled effect sizes for growth performance metrics (ADG, ADFI, and final BW), meta-regression analyses and subgroup analyses were conducted. The results indicate that treatment duration did not significantly moderate the pooled effect sizes for any of the three outcomes (ADG, *p* = 0.768; ADFI, *p* = 0.279 and final BW, *p* = 0.506 (Table S3.3.1)). Furthermore, the inclusion of this covariate did not substantially reduce the residual heterogeneity for each metric, suggesting that treatment duration could not explain the heterogeneity observed across studies. As shown in Figure S2A–C, subgroup analyses further showed that treatment duration did not significantly influence the overall pooled effect sizes for ADG, ADFI, or final BW. Moreover, the results indicate that initial BW did not significantly moderate the pooled effect sizes for ADG (*p* = 0.759), ADFI (*p* = 0.608). For final BW, the moderating effect of initial BW did not reach statistical significance (*p* = 0.068). Inclusion of this covariate resulted in limited reduction of residual heterogeneity for ADG (I^2^_res_ = 88.8%) and ADFI (I^2^_res_ = 85.6%), whereas a modest reduction was observed for final BW (I^2^_res_ = 80.0%). Subgroup analyses showed that initial BW significantly influenced the pooled effect size for final BW in <8 kg subgroup (SMD = −1.321, 95% CI: −1.645 to −0.998, *p* < 0.001), which exhibited no heterogeneity (I^2^ = 0.0%), but not in subgroup 2 (SMD = −0.730, 95% CI: −1.304 to −0.157, *p* = 0.013; I^2^ = 88.6%) (Figure S2D–F).

#### 3.1.3. Intestinal Morphology

**Figure 4:**
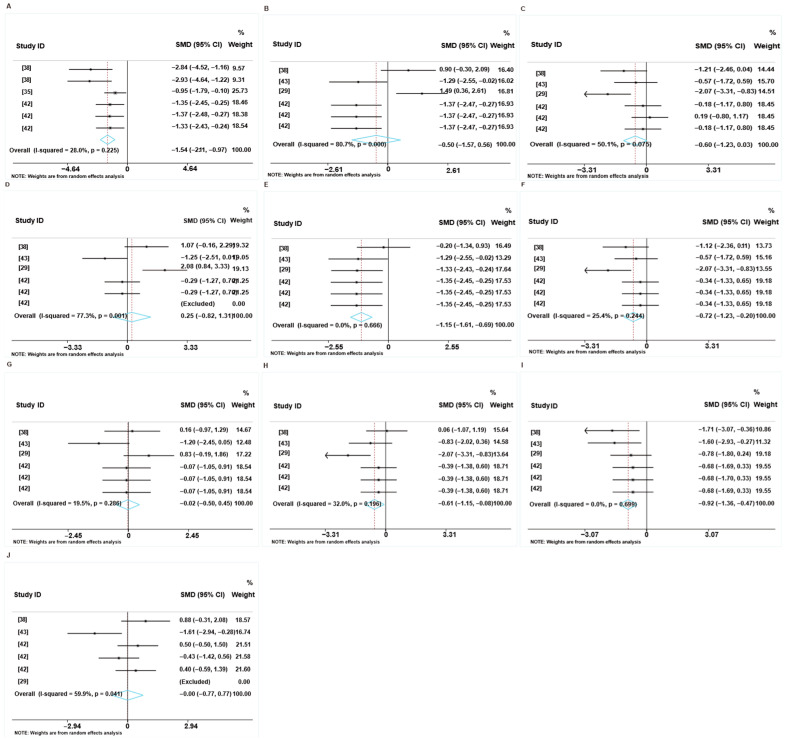
Effects of LP diets on the intestinal morphology of weaned piglets. The fecal consistency (**A**), VH (**B**), CD (**C**), and V:C ratio (**D**) in the duodenum. The VH (**E**), CD (**F**), and V:C ratio (**G**) in jejunum. The VH (**H**), CD (**I**), and V:C ratio (**J**) in the ileum. Control group = normal protein diet; experimental group = LP diet; SMD = standard mean difference; 95%CI = 95% confidence interval; I^2^ (0~100%) = test for heterogeneity. Test for overall effect: *p* value (significance level: *p* < 0.05). Black diamonds (♦) on the positive quadrant of the X-axis indicate an increase in the growth parameters, whereas those on the negative quadrant indicate a decrease.

Figure 4 shows the overall effects of the LP diets on the intestinal morphology of weaned piglets. Based on the dietary protein levels used in the included studies, the CP levels in the control groups ranged from 23.1% to 18.9%, while those in the LP diet groups ranged from 17.2% to 17.0%. The difference in protein levels between the two groups ranged from −5.9% to −1.7%. Specifically, the forest plot showed that the LP diet reduced the fecal consistency of weaned piglets (SMD: −1.54, 95% CI: −2.11 to −0.97, *p* < 0.05) compared to the control group, with low heterogeneity (I^2^ = 28%) (Figure 4A and Table S3.1.1). However, the LP diets did not affect the duodenum VH (SMD: −0.50, 95%CI: (−1.57 to 0.56), *p* > 0.05, I^2^ = 80.7%), CD (SMD: −0.60, 95%CI: (−1.23 to 0.03), *p* > 0.05, I^2^ = 50.1%) or V:C ratio (SMD: 0.25, 95%CI: (−0.82 to 1.31), *p* > 0.05, I^2^ = 77.3%), with high heterogeneity (Figure 4B–D and Table S3.1.1). The low-CP diet reduced jejunum VH (SMD: −1.15, 95%CI: (−1.61 to −0.69), *p* < 0.05, I^2^ = 0%) and CD (SMD: −0.72, 95%CI: (−1.23 to −0.20), *p* < 0.05, I^2^ = 25.4%), but did not affect the V:C ratio (SMD: −0.02, 95%CI: (−0.50 to 0.45), *p* > 0.05, I^2^ = 19.5%) (Figure 4E–G and Table S3.1.1). Furthermore, the LP diets reduced ileum VH (SMD: −0.61, 95%CI: (−1.15 to −0.08), *p* < 0.05, I^2^ = 32%) and CD (SMD: −0.92, 95%CI: (−1.36 to −0.47), *p* < 0.05, I^2^ = 0%), but had no effect on the V:C ratio (SMD: 0.00, 95%CI: (−0.77 to 0.77), *p* > 0.05, I^2^ = 59.9%) (Figure 4H–J and Table S3.1.1). To assess whether any single study unduly influenced the pooled results, we performed leave-one-out sensitivity analyses. These analyses showed that omitting any individual study from each subset did not materially change the pooled estimates, confirming the robustness of the findings (Tables S3.2.6–S3.2.15).

### 3.2. Effects of Feed Additives on the Growth Performance and Intestinal Morphology of Weaned Piglets

#### 3.2.1. Growth Performance

**Figure 5:**
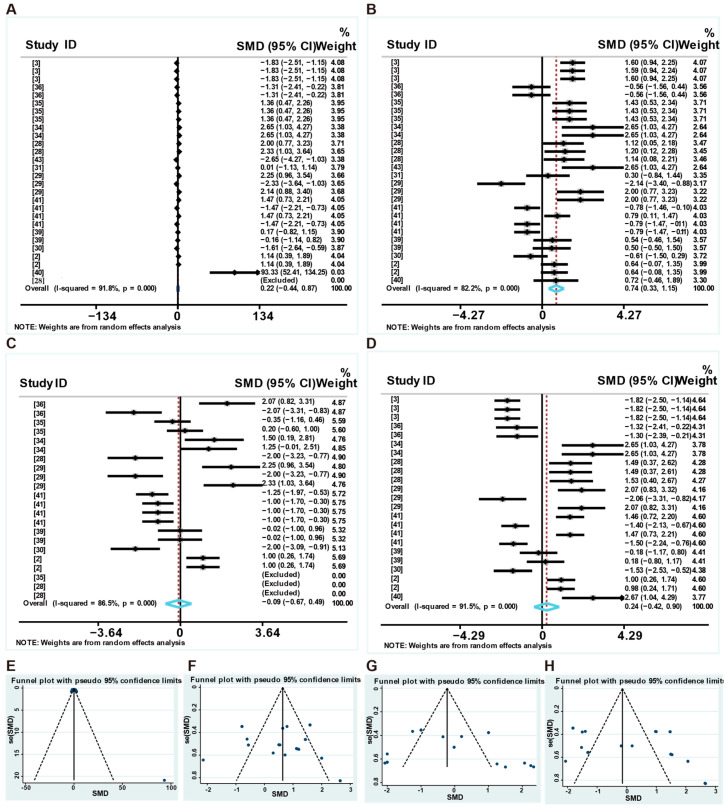
Forest plot of the effects of LP diets supplemented with feed additives on growth performance of weaned piglets. Forest plot of ADG (**A**), ADFI (**B**), G: F (**C**), and final BW (**D**) in weaned piglets. Funnel plot of ADG (**E**), ADFI (**F**), G: F (**G**), and final BW (**H**) in weaned piglets. Control = normal protein diet; experimental = LP diet; SMD = standard mean difference; 95%CI = 95% confidence interval; I^2^ (0~100%) = test for heterogeneity. Test for overall effect: *p* value (significance level: *p* < 0.05). Diamonds on the positive quadrant of the X-axis indicate an increase in the growth parameters, whereas those on the negative quadrant indicate a decrease.

Figure 5 shows the overall effects of feed additives on the growth performance of weaned piglets fed an LP diet. Specifically, the forest plot shows that feed additives had no effect on ADG (SMD: 0.216, 95% CI: −0.436 to 0.868, *p* = 0.516) compared to the control group, with high heterogeneity (I^2^ = 91.8%) (Figure 5A and Table S4.1.1) and significant publication bias (*p* = 0.046) (Figure 5E and Table S4.1.1). However, dietary supplements increased ADFI (SMD: 0.740, 95%CI: (0.332 to 1.1447), *p* < 0.001), with high heterogeneity (I^2^ = 82.2%) (Figure 5B and Table S4.1.1). The funnel plots and Egger linear regression results indicate no significant publication bias (*p* = 0.279) (Figure 5F and Table S4.1.1). As shown in Figure 5C, feed additives had no effect on the gain-to-feed ratio (SMD: −0.088, 95%CI: (−0.670 to 0.494), *p* = 0.766), with high heterogeneity (I^2^ = 86.5%). However, the test for publication bias in the gain-to-feed ratio yielded a nonsignificant result (*p* = 0.303) (Figure 5G and Table S4.1.1). In addition, supplementation with feed additives had no significant effect on the final BW of weaned piglets (SMD: 0.24, 95%CI: (−0.42 to 0.90), with high heterogeneity (I^2^ = 91.5%) (Figure 5D and Table S4.1.1), and significant publication bias (*p* = 0.026) (Figure 5H and Table S4.1.1). The leave-one-out sensitivity meta-analysis demonstrated that omitting any single study did not alter the nonsignificant finding, further supporting the robustness of the pooled estimates (Tables S4.2.1–S4.2.5). The trim-and-fill method revealed 6 missing observations for ADG and 10 missing observations for ADFI in the funnel plot (Figure S3).

#### 3.2.2. Subgroup Analysis for Growth Performance

**Figure 6:**
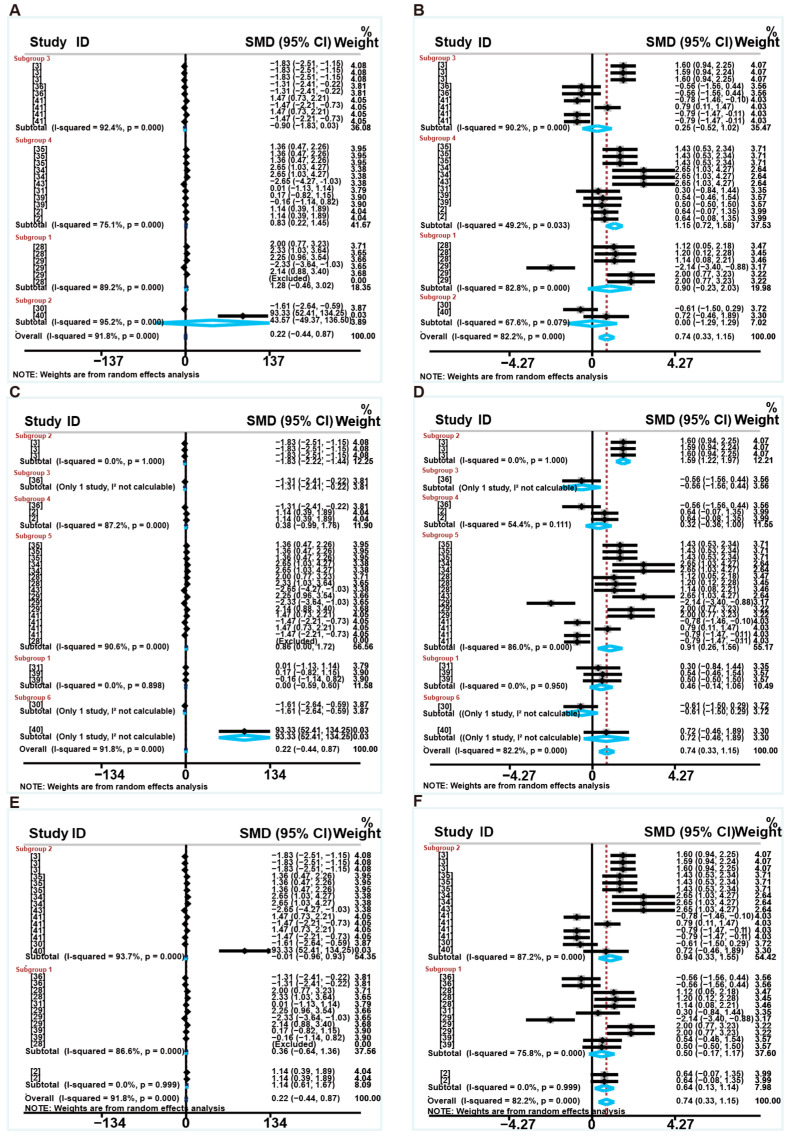
Subgroup analysis of the effects of protein levels, different types of feed additives, and weaning age on the growth performance in weaned piglets. Subgroup analysis of the effects of protein levels on ADG (**A**) and ADFI (**B**) in weaned piglets (subgroup 1 is 14% CP; subgroup 2 is 15% CP; subgroup 3 is 16% CP; subgroup 4 is 17% CP). Subgroup analysis of the effects of different types of feed additives on ADG (**C**) and ADFI (**D**) in weaned piglets (subgroup 1: plant extracts; subgroup 2: fatty acids; subgroup 3: vitamins; subgroup 4: enzymes; subgroup 5: AAs; subgroup 6: fermentable additives). Subgroup analysis of weaning age on ADG (**E**) and ADFI (**F**) in weaned piglets (subgroup 1 is “<21 days”; subgroup 2 is “≥21 days”). Control group = LP diets; experimental group = LP diet + feed additives; SMD = standard mean difference; 95%CI = 95% confidence interval; I^2^ (0~100%) = test for heterogeneity. Test for overall effect: *p* value (significance level: *p* < 0.05). Diamonds on the positive quadrant of the X-axis indicate an increase in the growth parameters, whereas those on the negative quadrant indicate a decrease.

These experiments included different levels of protein reduction (13%, 14%, 15%, 16%, and 17%), different types of feed additives (plant extracts, fatty acids, vitamins, enzymes, AAs, and carbohydrates), different weaning ages (<21 days, =21 days, and >21 days), different treatment durations (<4 weeks and ≥4 weeks) and different initial BW (<8 kg and ≥8 kg). Experimental differences may affect heterogeneity and results, so we subdivided the subgroups into different levels of dietary protein reduction, types of feed additives, weaning age, treatment duration and initial BW (Figure 6, Figures S4 and S5 and Table S4.3.1). We further explored sources of heterogeneity and factors influencing results.

Figure 6A,B show the effects of different levels of protein reduction on the growth performance of weaned piglets fed LP diets supplemented with feed additives. Subgroups 1–4 respectively correspond to 14% CP to 17% CP. Compared to the LP diets, subgroups comprising 17% supplemented with feed additives increased the ADG (SMD: 0.835, 95%CI: 0.216 to 1.454, *p* = 0.008), and grouping factors were not a source of heterogeneity (Figure 6A). Similarly, compared to the LP diets, subgroups comprising 17% supplemented with feed additives increased the ADFI (SMD: 1.150, 95%CI: 0.720 to 1.581, *p* < 0.001), with lower heterogeneity (I^2^ = 49.2%) (Figure 6B). Therefore, we next assessed the different types of feed additives and conducted subgroup analyses.

We divided the subgroups according to the different types of feed additives and examined the sources of heterogeneity and their effects on the results. As shown in Figure 6C,D, compared with the LP diets, subgroups supplemented with feed additives comprising fatty acids (subgroup 2) and vitamins (subgroup 3) reduced the ADG (*p* < 0.05) (Figure 6C), and grouping factors were not a source of heterogeneity. In addition, the subgroups supplemented with fatty acids (subgroup 2) and AAs (subgroup 5) showed a significant increase in weaned piglets’ ADFI compared to the LP diets (*p* < 0.05), with most of the heterogeneity attributed to the AA subgroup (subgroup 5) (I^2^ = 86%) (Figure 6D).

As shown in Figure 6E,F, compared to the LP diets, none of the subgroups had a significant effect on the ADG of weaned piglets, with high-level heterogeneity. Meanwhile, as presented in Figure 6F, the ≥21 days subgroups showed a significant increase in the ADFI of weaned piglets compared to those fed the LP diets (*p* < 0.001) (I^2^ = 87%).

In addition, meta-regression analyses were conducted to further investigate the source of heterogeneity. The results demonstrate that neither CP level nor weaning age was responsible for the high heterogeneity in ADG and ADFI (Table S4.1.1) (*p* > 0.05). However, the type of additive may be responsible for the high heterogeneity in ADG (Table S4.1.1) (*p* = 0.033).

The meta-regression and subgroup analyses revealed that treatment duration, as a covariate, had no significant effect on ADG (*p* = 0.138) or the G:F ratio (*p* = 0.709) (Table S4.3.1). However, a significant effect was observed on ADFI (*p* = 0.048) (Table S4.3.1), with a larger effect size in the <4 weeks subgroup (SMD = 1.497, *p* < 0.001) compared to the ≥4 weeks subgroup (SMD = 0.501, *p* = 0.041), suggesting that a shorter treatment duration (<4 weeks) was more effective in increasing ADFI (Figure S4B and Table S4.3.1). In addition, treatment duration significantly influenced final body weight (*p* = 0.006) (Table S4.3.1). The <4 weeks subgroup showed a significant positive effect (SMD = 1.784, *p* < 0.001), whereas no significant effect was observed in the ≥4 weeks subgroup (SMD = −0.336, *p* = 0.354) (Figure S4D and Table S4.3.1). This finding indicates that a shorter treatment duration (<4 weeks) improved the final body weight of weaned piglets.

When evaluating the covariate initial BW, the analysis revealed that initial body weight significantly modulated the effect on ADG (*p* = 0.020) (Table S4.3.1). In weaned piglets with a lower initial BW (<8 kg), a significant positive effect was observed (SMD = 0.835, *p* = 0.021), whereas no significant effect was detected in piglets with a higher initial BW (≥8 kg) (*p* = 0.057) (Figure S5A and Table S4.3.1). For ADFI (*p* = 0.375), G:F ratio (*p* = 0.732) and final BW (*p* = 0.078), initial BW did not significantly influence the effect (Figure S4B and Table S4.3.1).

#### 3.2.3. Intestinal Morphology

**Figure 7:**
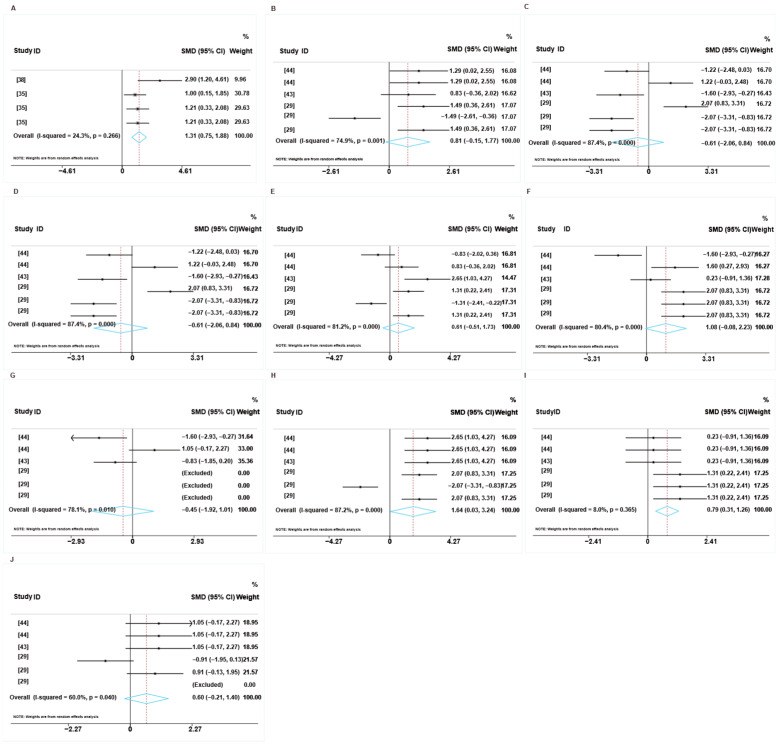
Cumulative probabilities of LP diets supplemented with feed additives on the intestinal morphology in weaned piglets. (**A**) The fecal consistency of weaned piglets. The VH (**B**), CD (**C**), and V:C ratio (**D**) in duodenum. The VH (**E**), CD (**F**), and V:C ratio (**G**) in jejunum. The VH (**H**), CD (**I**), and V:C ratio (**J**) in ileum. Control group = LP diet; experimental group = LP diets + feed additives; SMD = standard mean difference; 95%CI = 95% confidence interval; I^2^ (0~100%) = test for heterogeneity. Test for overall effect: *p* value (significance level: *p* < 0.05). Black diamonds (♦) on the positive quadrant of the X-axis indicates an increase in the growth parameters, whereas those on the negative quadrant indicate a decrease.

The results of the forest plots suggest that the feed additives led to a significant increase in the fecal consistency of weaned piglets fed the LP diet (SMD: 1.31, 95% CI: 0.75 to 1.88, *p* < 0.05, I^2^ = 24.3%) (Figure 7A). However, as shown in Figure 7B–D, supplementation with feed additives had no effect on the duodenum VH, CD, or V:C compared to the LP diets (*p* > 0.05). In addition, the feed additives had no significant effects on the VH, CD, or V:C in the jejunum of weaned piglets compared to the LP diets (*p* ≥ 0.05) (Figure 7E–G). Furthermore, the feed additives increased ileum VH (SMD: 1.64, 95%CI: (0.03 to 3.24), *p* < 0.05, I^2^ = 87.2%) and CD (SMD: 0.79, 95%CI: (0.31 to 1.26), *p* < 0.05, I^2^ = 8.0%), but had no effect on the V:C ratio (*p* > 0.05) (Figure 7H–J). Based on the dietary protein levels used in the included studies, the CP levels used in the LP diet groups and feed additive groups were analyzed. These analyses show that omitting any individual study from each subset did not materially change the pooled estimates, confirming the robustness of the findings (Tables S4.2.6–S4.2.15).

#### 3.2.4. Network Meta-Analysis to Evaluate Optimal Feed Additives

**Figure 8:**
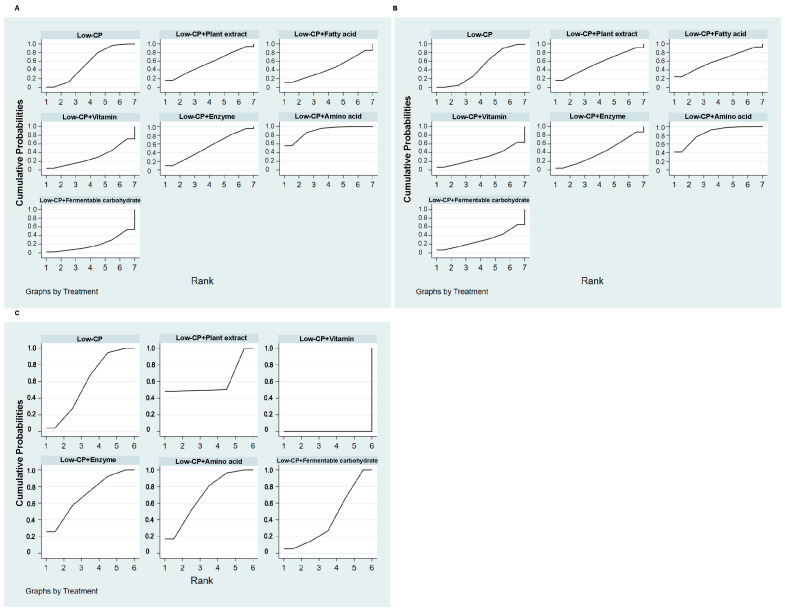
Feed additives assessment. (**A**) Surface under the cumulative ranking curve for ADG; (**B**) surface under the cumulative ranking curve for ADFI; (**C**) surface under the cumulative ranking curve for gain-to-feed ratio.

Figure 8 shows the feed additive assessment score based on network meta-analysis (NMA) (Table S4.4.1). In terms of improving ADG, the amino-terminal intervention demonstrated the highest likelihood of being the most effective, as evidenced by SUCRA ranking results (AA (56%) > plant extracts (15.7%) > fatty acids or enzymes (10.2%) > vitamins (5%) > fermentable carbohydrates (2.4%)). The results show that AAs can be considered the main feed additives, followed by plant extracts, improving ADG (Figure 8A) and ADFI (AAs (42.9%) > fatty acids (24.3%) > plant extracts (14.9%) > vitamins (6.4%) > fermentable carbohydrates (6.2%) > enzymes (5.2%)) (Figure 8B) in weaned piglets fed an LP diet. Concerning the gain-to-feed ratio, plant extracts demonstrated superior performance to AAs (plant extracts (47%) > enzymes (26.2%) > AAs (18%) > fermentable carbohydrates (4.9%)) (Figure 8C).

## 4. Discussion

### 4.1. Effects of LP Diets on Weaned Piglets: Growth Suppression and Intestinal Health Improvements

With the development of large-scale pig production driving increasing precision in feed formulation, compared with the NRC 1998 [45], the NRC 2012 [46] introduced the concept of standardized ileal digestible (SID) AA requirements. The current Chinese national standard for GB/T 39235-2020 [47]“Nutritional Requirements for Pigs” specifies the nutrient requirements for lean-type pigs, recommending a CP level of 21% for piglets weighing 3–8 kg. Analysis of several nutritional standards reveals that recommended dietary protein levels have consistently decreased, and compared with NRC 2012 [46], GB/T 39235-2020 showed a further reduction in protein recommendations. This aligns with the developmental needs to reduce nitrogen emissions [4,12,35,48,49,50], improve nitrogen utilization [44], advance pig production, and deepen animal nutrition research. In previous studies, dietary CP reductions of more than 2 percentage points compared with the control diet were generally considered LP diets. However, reports on the efficacy of LP diets have been inconsistent, making the threshold for protein reduction critically important. Nevertheless, due to global climate, regional, and feed ingredient variations, there is currently no single, universally accepted standard for defining an LP diet.

This study integrated 19 studies with 23 datasets and employed meta-analysis to reveal the significant growth-suppressing effects of LP diets (below 18%) in weaned piglets. The results show that LP diets led to a significant decrease in average daily gain, feed efficiency, and final body weight, accompanied by an increase in the feed-to-gain ratio. Although these findings differ from some studies reporting positive effects under specific conditions [51], our summary effect sizes (SMD for ADG: −1.322) indicate that reducing dietary CP levels below 18% constrains the growth potential of weaned piglets, even with essential AA supplementation, without additional interventions. Subgroup analysis further revealed that different levels of protein reduction (13–17% CP) exerted varying inhibitory effects on ADG. Similarly, one study using a broken-line model to analyze the relationship between dietary CP level and growth performance in nursery pigs found that ADG decreased when dietary CP fell below 18.4% (95% CI: 16.3–18.4). The G:F ratio decreased when dietary CP fell below 18.3% (95% CI: 17.4–19.2). Notably, all growth performance indicators analyzed in this study exhibited high heterogeneity (I^2^ > 75%), indicating substantial variation in true effects across studies. However, subgroup analysis and meta-regression failed to attribute this heterogeneity to single factors, such as dietary CP reduction level, weaning age, treatment duration, or initial body weight. This suggests that the sources of heterogeneity may be more complex, potentially related to differences in dietary AA balance patterns, energy levels, ingredient composition, or housing conditions across studies. For instance, if the AA profile of an LP diet does not precisely match the “ideal protein” pattern for piglets, growth performance could vary considerably even at similar protein levels [52]. Therefore, further investigation into heterogeneity through expanded sample sizes or more refined subgroup criteria is needed.

Further analysis revealed that compared with control diets, LP diets (CP < 18%) reduced the diarrhea incidence in weaned piglets. Notably, this conclusion is based on a protein level difference between the two groups in this model ranging from −5.9% to −1.7%. This finding is consistent with most studies. The underlying mechanism may be related to a reduction in undigested protein in the diet, thereby decreasing the nitrogen source required for the proliferation of pathogenic bacteria in the intestine, maintaining gut microbiota balance and barrier function [6,53,54]. This provides strong evidence for the application of LP diets in alleviating diarrhea in weaned piglets. However, some studies have shown that reducing dietary protein levels below 18% can affect intestinal development in weaned piglets [55]. Additionally, lowering dietary protein levels can induce an increase in intestinal crypt depth, leading to small intestinal atrophy and impairing nutrient digestion and absorption [42]. In this analysis, LP diets were found to decrease villus height and crypt depth in the jejunum and ileum. Previous research has indicated that LP diets can reduce the expression levels of the barrier factor zonula occludens-1 (ZO-1) and the stem cell proliferation factor leucine-rich repeat-containing G protein-coupled receptor 5 (Lgr5) in the jejunum [56]. In summary, appropriately reducing dietary CP levels can reduce the diarrhea incidence in piglets and enhance intestinal homeostasis. Improved LP diet technologies hold promises for broader application in the swine industry.

### 4.2. Nutritional Intervention Strategies: Key to Mitigating the Negative Effects of LP Diets

To address the growth suppression associated with LP diets, this study systematically evaluated the efficacy of various feed additives for the first time. Overall, when dietary CP levels were below 18%, additives did not significantly improve ADG or the G: F ratio but increased average daily feed intake. This finding suggests that additives may stimulate feed intake by improving palatability or modulating satiety centers. However, the increase in feed intake did not directly translate into efficient weight gain, likely due to the relative deficiency of substrates (AAs) required for protein synthesis.

Subgroup analysis further revealed the effects of different additives and experimental conditions. Supplementation with additives in diets with 17% CP increased ADG and ADFI, indicating that additives can better exert their synergistic effects when protein reduction is modest. Additionally, meta-regression confirmed that additive type was a significant source of heterogeneity for ADG. Subsequent network meta-analysis quantified the comparative efficacy of different additives, clearly establishing the dominant role of AA in improving ADG and ADFI. However, due to the varying doses and types of AA added in the included studies, we were unable to draw a unified conclusion on this issue. Nonetheless, we observed that within the AA subgroup, seven studies added only a single AA (L-isoleucine or L-valine), while seven studies added a combination of multiple AAs. Plant extracts performed best in improving feed efficiency (G: F ratio). The plant extracts included in this subgroup were glycerol monolaurate (2 kg/T) and phytogenic water additive (4 mL/L, 8 mL/L). This ranking provides direct evidence for optimizing additive formulations in LP diets for production practices. The prominent role of AAs is intrinsically linked to their function as substrates for protein synthesis; supplementing exogenous AAs under protein-restricted conditions is the most direct and effective means to compensate for endogenous AA deficiencies and support body protein deposition [57]. The effects of plant extracts may be more indirect, improving nutrient utilization efficiency by modulating gut microbiota, enhancing digestive enzyme activity, or exerting anti-inflammatory and antioxidant effects [58].

The study also identified treatment duration (<4 weeks) and initial piglet body weight (<8 kg) as significant moderators influencing the efficacy of additives. Supplementation was more effective when applied during the early post-weaning period (<4 weeks) or in low-body-weight piglets. This is likely because early-weaned piglets have a more immature digestive system and experience greater weaning stress, making them more responsive to exogenous nutritional interventions [6,53,54].

### 4.3. Limitations and Future Directions

Although this study provides valuable evidence-based insights, it has limitations. First, the quality assessment of included studies revealed that most had an “unclear” risk of bias, and significant publication bias was detected for some outcomes (ADG and final BW), potentially overestimating the magnitude of negative effects. While the trim-and-fill method was used for correction, the results should still be interpreted cautiously. Second, the high heterogeneity observed in the analyses was not fully explained. Future research should meticulously record and report factors that may influence outcomes, such as dietary net energy levels, AA balance patterns, anti-nutritional factors in ingredients, and the health status of piglets. Finally, this analysis focused solely on villus height and crypt depth for intestinal morphology. Future meta-analyses could integrate more data on intestinal barrier function, microbiome structure, and immune status to more comprehensively elucidate the mechanisms underlying the effects of LP diets and additives.

In summary, this meta-analysis clarifies the paradox of LP diets in weaned piglets, namely their ability to improve intestinal health while suppressing growth performance. The key to resolving this paradox lies in implementing precise nutritional interventions. In LP diets, supplementing with AAs and plant extracts, particularly during the early post-weaning period and in low-body-weight piglets, represents an effective strategy to mitigate growth suppression. Future research should focus on optimizing additive combinations, elucidating their synergistic mechanisms, and developing LP diet formulation technologies based on net energy systems and precise AA balance to facilitate their widespread application in swine production.

## 5. Conclusions

**Figure 9:**
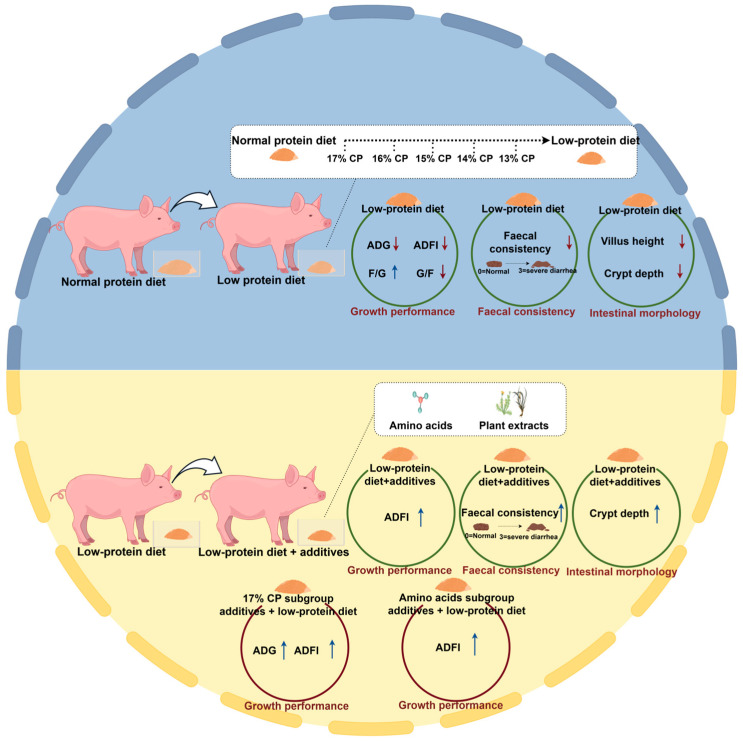
Summary of findings of meta-analysis. Red arrows indicate a decrease, and blue arrows indicate increase.

Our meta-analysis indicated that reductions in dietary CP levels above the 17% level reduce growth performance and intestinal morphology in weaned piglets. To address the effects of LP diets, it is now common to supplement LP diets with plant extracts, AAs, fatty acids, vitamins, enzymes, and carbohydrates. Among them, LP diets supplemented with AAs and plant extracts improve the growth performance and intestinal morphology of weaned piglets, compensating for the effects of LP diets (Figure 9). In addition, we reviewed recent years of research on LP diets. Feeding LP diets to weaned piglets has significant advantages in reducing nitrogen emissions and lowering the diarrhea incidence. However, feeding LP diets to weaned piglets reduces growth performance and, to some extent, causes homeostatic imbalance in the gut and reduced productivity in pig farming. Therefore, three nutritional interventions are currently available, including the use of a net energy system to formulate LP diets, nutritional modulation to improve AA utilization, and optimization of feed formulations for diversification and feed processing. Further research on the digestible protein and AA requirements of weaned piglets within a net energy framework and a broader exploration of diet formulation diversification are needed in the future. Based on the results of the meta-analysis, the optimization of LP diets could focus on AAs and plant extracts.

## Data Availability

The datasets used and/or analyzed during the current study are available from the corresponding author on reasonable request.

## Supplementary Materials

The following supporting information can be downloaded at https://www.mdpi.com/article/10.3390/ani16081157/s1: File S1: PRISMA-2020 checklist [59]. File S2: Appendix to “Disadvantage in the use of low-protein diets in weaned pigs and nutritional interventions: A meta-analysis”. File S3: Supplementary methods for map drawing. File S4: Supplementary methods for trim-and-fill analysis.

## Abbreviations

The following abbreviations are used in this manuscript:

AA	Amino acids
ADG	Average daily gain
ADFI	Average daily feed intake
CD	Crypt depth
CI	Confidence interval
CP	Crude protein
SMD	Standard mean difference
V:C ratio	Villus height: crypt depth ratio
VH	Villus height
