# Supplementary material for: Disadvantages of the Use of Low-Protein Diets in Weaned Piglets and Nutritional Interventions: A Meta-Analysis
Source: Animals (Basel). 2026 Apr 10;16(8):1157. doi: 10.3390/ani16081157 (PMC13112980; doi:10.3390/ani16081157)
Supplement: Supplementary file 1 [file animals-16-01157-s001.zip › S2.Appendix to meta-analysis.pdf]

Appendix to "Disadvantage in the use of low-protein diets in weaned pigs and nutritional interventions: A meta-analysis"

Jingchun Gao[a], Xiaoyi Long[a], Qingsong Tang, Haiyang Wei, Xie Peng, Yetong Xu, Zhiru Tang and Zhihong Sun[*]

In this Appendix we provide additional information on the analyses we described in the manuscript titled "Disadvantage in the use of low-protein diets in weaned pigs and nutritional interventions: A meta-analysis".

Table of Contents

Appendix to "Disadvantage in the use of low-protein diets in weaned pigs and nutritional interventions: A meta-analysis" 1

1 Coded Variables and Descriptives 2

1.1 Search Strategy and Study Characteristics (Table S1.1.1 – 1.1.3) 2

1.2 Geographic Distribution of Included Studies (Figure S1) 11

2 Study Quality Assessment 12

2.1 Study quality assessment (Table S2.1.1) 12

3 Impact of Low Protein Diets on Growth Performance and Intestinal Morphology 14

3.1 Main Meta-Analysis Results (Table S3.1.1) 14

3.2 Sensitivity Analysis (Table S3.2.1 - 3.2.15) 16

3.3 Subgroup Analyses by CP Level, Weaning Age, treatment duration and initial BW (Table S3.3.1) 34

3.4 Effects of low-protein diets on growth performance of weaned piglets: subgroup analyses by treatment duration and initial body weight (Figure S2) 36

3.5 Trim-and-fill funnel plot for the effects of low-protein diets on ADG and ADFI in weaned piglets. 38

4 Impact of Feed Additives on Growth Performance and Intestinal Morphology 39

4.1 Main Meta-Analysis Results (Table S4.1.1) 39

4.2 Sensitivity Analysis (Table 4.2.1-4.2.15) 42

4.3 Subgroup Analyses by Additive Type, CP Level, Weaning Age, treatment duration and initial BW (Table 4.3.1) 61

4.4 Effect of feed additives on growth performance of weaned piglets fed low-protein diets: subgroup analyses by treatment duration (Figure S4) 64

4.5 Effect of feed additives on growth performance of weaned piglets fed low-protein diets: subgroup analyses by initial body weight (Figure S5) 65

4.6 Trim-and-fill funnel plot for the effect of feed additives on ADG and ADFI in weaned piglets fed low-protein diets 66

4.7 Efficacy Ranking of Nutritional Interventions (Table S4.4.1) 67

References 68

1 Coded Variables and Descriptives

1.1 Search Strategy and Study Characteristics (Table S1.1.1 – 1.1.3)

Table S1.1.1 Search strategy

Search	Query	Items found
Science Direct		
#1	Search: Low protein AND piglets; Filters: Research articles	11405
#2	Search: Title, abstract, keywords: Low protein AND piglets	268
#3	Search: low protein AND weaned pigs; Filters: Research articles	4547
#1 AND #2 AND #3		191
Web of Science		
#1	Search: pig OR piglets OR weaned pigs (Abstract)	327527
#2	Search: low protein (Abstract)	833274
#3	Search: low protein (Title)	20635
#1 AND #2 AND #3		388
#4	Search: low protein (Topic) and weaned pigs (Topic) and piglets (Topic) and pig (Topic) and low protein (Abstract) and weaned pigs (Abstract)	837
PubMed		
#1	Search: (pig [Title/Abstract] OR piglets [Title/Abstract] OR weaned pigs [Title/Abstract])	160231
#2	Search: (low protein [Title/Abstract])	9743
#1 AND #2		218
#3	(low protein [Title/Abstract]) AND (piglets [Title/Abstract])	86

Table S1.1.2 Characteristics of studies

Study	Country	Weaning age, d	Con CP, %	LP CP, %	Sample size	Initial BW, kg	CP level difference	Reference standard	Breed	Duration	Outcomes
[3]	China	28	20.00	16.00	24	7.93±0.70	-4.00 %	NRC 2012	Landrace × Large White × Duroc	4 weeks	ADG, ADFI, F:G, Final BW, Plasma cytolines.
[33]	China	21	20.00	16.00	25	6.39±0.02	-4.00 %	NRC 2012	Duroc × Landrace × York × shire	14 day	ADG, ADFI, G:F, Final BW, Diarrhea incidence, Body composition, Serum biochemical parameters.
[36]	America	21	20.00	17.00	32	8.80±0.05	-3.00 %	NRC 2012	Duroc × Yorkshire × Landrace	28 day	ADG, ADFI, G:F, Final BW, Apparent total tract digestibility.
[35]	Portugal	28	20.00	17.00	12	6.92±0.61	-3.00 %	NRC 1998	Landrace × Duroc	4 weeks	ADG, ADFI, F:G,Nitrogen balance.
[5]	China	28	18.83	13.05	18	6.47±0.04	-5.82 %	-	-	14 day	ADG, ADFI, F:G.
[34]	China	28	20.00	17.00	6	8.45±0.56	-3.00 %	NRC 2012	Duroc × Landrace × Large White	14 day	ADG, ADFI, G:F, Final BW.

[6]	Canada	18±1	21.00	17.00	24	6.20±0.10	-4.00 %	NRC 1998	Cotswold	21 day	ADG, ADFI, G:F, Final BW, Small intestine morphology .
[28]	America	21	20.00	14.00	8	6.14	-6.00 %	NRC 2012	Duroc sire line × Large White × Landrace dam	21 day	ADG, ADFI, G:F, Final BW.
[32]	China	21	20.70	16.70	6	8.24±0.67	-4.00 %	NRC 1998	Duroc × Landrace × Yorkshire	14 day	ADG, ADFI, F:G, Final BW.
[32]	China	21	20.70	12.70	6	8.24±0.67	-8.00 %	NRC 1998	Duroc × Landrace × Yorkshire	14 day	ADG, ADFI, F:G, Final BW.
[43]	China	28	20.90	17.10	36	7.97±0.11	-3.90 %	NRC 1998	Large White × Landrace × Duroc	14 day	ADG, ADFI, F:G,Small intestine morphology.
[38]	Germany	17±2	21.00	17.00	24	6.44±0.14	-3.00 %	NRC 1998	Duroc × Yorkshire × Landrace	3 weeks	ADG, ADFI, F:G, Final BW, Small intestine morphology.
[31]	China	21	18.80	17.20	18	6.00±0.15	-1.60 %	NRC 2012	Duroc × Landrace × Large Yorkshire	14 day	ADG, ADFI, F:G.
[29]	America	21	20.00/ 19.00	14.00/ 13.00	8	6.75±0.15	-6.00 %	NRC 2012	Duroc sire line× Large White × Landrace dam	5 weeks	ADG, ADFI, G:F, Final BW, Small intestine morphology.
[41]	Netherlands	24±2	19.00	16.00	54	6.90±0.34	-3.00 %	NRC 2012	Hypor Libra × Maxter	27 day	ADG, ADFI, G:F, Final BW.

[39]	America	21	24.00	17.00	8	9.02±0.17	-7.00 %	NSNG V2.0	Duroc sire line × Large White × Landrace dam	42 day	ADG, ADFI, G:F, Final BW.
[42]	China	18±1	23.10	17.20	8	6.80	-5.90 %	NRC 1998	Large White × Landrace barrows	14 day	ADG, ADFI, F:G, Final BW, Small intestine morphology.
[42]	China	18±1	21.20	17.20	8	6.80	-4.00 %	NRC 1998	Large White × Landrace barrows	14 day	ADG, ADFI, F:G, Final BW, Small intestine morphology.
[42]	China	18±1	18.90	17.20	8	6.80	-1.70 %	NRC 1998	Large White × Landrace barrows	14 day	ADG, ADFI, F:G, Final BW, Small intestine morphology.
[30]	Netherlands	26±0.8	22.00	15.00	10	8.70±0.17	-7.00 %	CVB 2003	Yorkshire × Landrace	28 day	ADG, ADFI, G:F, Final BW.
[40]	China	28	18.83	13.05	18	6.47±0.04	-5.78 %	NRC 1998	Duroc × Yorkshire-Landrace	14 day	ADG, ADFI, F:G, Ileal intestine morphology.
[2]	Korea	-	19.00/ 18.05	17.00/ 16.15	16	6.99±0.21	-2.00 %/-1.9 %	-	Yorkshire × Landrace × Duroc	4 weeks	ADG, ADFI, G:F, Final BW.
[37]	Denmark	28	19.20/ 18.90	17.60/ 17.40	141/65	5.50~9.00	-1.60 %/-1.5 %	Danish Nutrient Requirement Standards	Duroc × Danish Landrace × Danish Yorkshire	10 day	ADG, Final BW.

[37]	Denmark	28	19.20/ 18.90	17.60/ 17.40	141/56	5.50~9.00	-1.60 %/- 1.5 %	Danish Nutrient Requirement Standards	Duroc × Danish Landrace × Danish Yorkshire	10 day	ADG, Final BW.
[37]	Denmark	28	19.20/ 18.90	15.40/ 15.10	141/17	5.50~9.00	-3.8 %	Danish Nutrient Requirement Standards	Duroc × Danish Landrace × Danish Yorkshire	10 day	ADG, Final BW.

CP, Crude Protein; BW, body weight; Final BW, Final body weight.; ADG, Average daily gain; ADFI, Average daily feed intake; F: G, feed: gain ratio; G: F, gain: feed ratio; VC, Villus height: Crypt depth; NRC 1998, Nutrient requirements of swine (10th rev. ed.); CVB 2003 Centraal Veevoederbureau, (2003).

Table S1.1.3 Characteristics of studies

Study	Country	Weaning age, d	LP CP, %	LP+Additive CP, %	Sample size	Treatments	Initial BW, kg	Duration or final BW	Outcomes
[3]	China	28	16.00	16.00	24	sodium butyrate (0.2%)	7.93±0.70	4 weeks	ADG, ADFI, F/G, Plasma cytolines
[3]	China	28	16.00	16.00	24	medium-chain fatty acids (0.2%)	7.93±0.70	4 weeks	ADG, ADFI, F/G, Plasma cytolines
[3]	China	28	16.00	16.00	24	n-3 polyunsaturated fatty acids (0.2%)	7.93±0.70	4 weeks	ADG, ADFI, F/G, Plasma cytolines
[36]	America	21	16.00	16.00	32	myo-inositol(2g/kg)	8.80±0.05	28 days	ADG, ADFI, G:F, Final BW, Apparent total tract digestibility.
[36]	America	21	16.00	16.00	32	Phytase(3000FTU/kg)	8.80±0.05	28 days	ADG, ADFI, G:F, Final BW, Apparent total tract digestibility.
[35]	Portugal	28	17.00	17.00	12	Ile (0.13%)	6.92±0.61	4 weeks	ADG, ADFI, F:G, nitrogen balance
[35]	Portugal	28	17.00	17.00	12	Val (0.15%)	6.92±0.61	4 weeks	ADG, ADFI, F:G, nitrogen balance
[35]	Portugal	28	17.00	17.00	12	Ile+Val (0.13%+0.15%)	6.92±0.61	4 weeks	ADG, ADFI, F:G, nitrogen balance
[34]	China	28	17.00	17.00	6	Leu(0.24%)+Ile(0.17%)+ Val(0.16%)	8.45±0.56	14 day	ADG, ADFI, G:F, Final BW.

[34]	China	28	17.00	17.00	6	Leu(0.48%)+Ile(0.34%)+ Val(0.32%)	8.45±0.56	14 day	ADG, ADFI, G:F, Final BW.
[28]	America	21	14.00	14.00	8	Val(0.48%)	6.14	21 day	ADG, ADFI, G:F, Final BW.
[28]	America	21	14.00	14.00	8	Ile(0.33%)	6.14	21 day	ADG, ADFI, G:F, Final BW.
[28]	America	21	14.00	14.00	8	Val(0.48%)+Ile(0.33%)	6.14	21 day	ADG, ADFI, G:F, Final BW.
[44]	China	21	17.00	17.00	6	Lys, Trp, Thr, Leu, Ile, Val, Met and Cys(10%)	8.15±1.10	4 weeks	Small intestine morphology.
[44]	China	21	17.00	17.00	6	SID Met + Cys、Thr and Trp(12%)	8.15±1.10	4 weeks	Small intestine morphology.
[43]	China	28	17.10	17.10	36	supplementing Leu, Ile and Val to 17.9 % crude protein	7.97±0.11	14 day	ADG, ADFI, F:G,Small intestine morphology(n=6).
[31]	China	21	17.20	17.20	18	2 kg/T glycerol monolaurate	6.00±0.15	14 day	ADG, ADFI, F:G,
[29]	America	21	14.00/13.00	14.00/13.00	8	Val (0.31%)	6.75±0.15	5 weeks	ADG, ADFI, G:F, Final BW,Small intestine morphology.

[29]	America	21	14.00/13.00	14.00/13.00	8	Ile (0.30%)	6.75±0.15	5 weeks	ADG, ADFI, G:F, Final BW,Small intestine morphology.
[29]	America	21	14.00/13.00	14.00/13.00	8	Val (0.31%) and Ile (0.30%)	6.75±0.15	5 weeks	ADG, ADFI, G:F, Final BW,Small intestine morphology.
[41]	Netherlands	24±2	16.00	16.00	54	AA profile as the PC diet	6.90±0.34	27 day	ADG, ADFI, G:F, Final BW.
[41]	Netherlands	24±2	16.00	16.00	54	His, Thr, Trp, Met+Cys	6.90±0.34	27 day	ADG, ADFI, G:F, Final BW.
[41]	Netherlands	24±2	16.00	16.00	54	10%Leu, Ile, and Val	6.90±0.34	27 day	ADG, ADFI, G:F, Final BW.
[41]	Netherlands	24±2	16.00	16.00	54	10%EAA except for Lys	6.90±0.34	27 day	ADG, ADFI, G:F, Final BW.
[39]	America	21	17.00	17.00	8	4mL/L phytogenic water additive	9.02±0.17	42 day	ADG, ADFI, G:F, Final BW.
[39]	America	21	17.00	17.00	8	8mL/L phytogenic water additive	9.02±0.17	42 day	ADG, ADFI, G:F, Final BW.
[30]	Netherlan	26±0.8	15.00	15.00	10	13.5% fermentable	8.70±0.17	28 day	ADG, ADFI, G:F, Final BW.

	ds					carbohydrates			
[2]	Korea	-	17.00/1<br>6.15	17.00/16.15	16	0.3% proteases	6.99±0.21	4 weeks	ADG, ADFI, G:F, Final BW.
[2]	Korea	-	17.00/1<br>6.15	17.00/16.15	16	0.5% proteases	6.99±0.21	4 weeks	ADG, ADFI, G:F, Final BW.

Ile, Isoleucine; Val, Valine; Leu, Leucine; Lys, Lysine; Trp, Tryptophan; Thr, Threonine; Met, Methionine; Cys, Cysteine; His, Histidine.

1.2 Geographic Distribution of Included Studies (Figure S1)

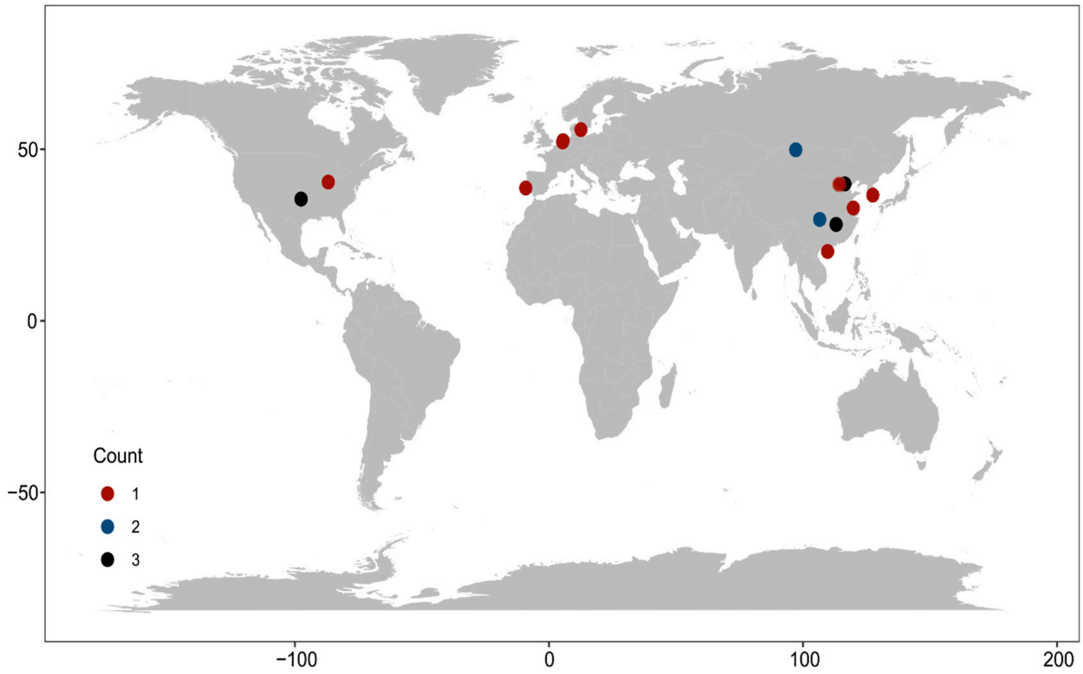

Figure S1. Distribution of study areas around the world. Two items overlapping study areas result in blue dots, and three items overlapping study areas result in black dots.

2 Study Quality Assessment

2.1 Study quality assessment (Table S2.1.1)

Table S2.1.1 Study quality assessment

Study	Random sequence generation	Allocation concealment	Blinding of participants and personnel	Blinding of outcome assessment	Incomplete outcome data	Selective reporting	Other bias	Quality
[3]	0	1	1	1	0	0	0	Low
[33]	0	1	1	1	0	0	0	Low
[36]	0	1	1	1	0	0	0	Low
[35]	0	1	1	1	0	0	0	Low
[5]	0	1	1	1	0	0	0	Low
[34]	0	1	1	1	0	0	0	Low
[6]	0	1	1	1	0	0	0	Low
[28]	0	1	1	1	0	0	0	Low
[32]	0	1	1	1	0	0	0	Low
[43]	0	1	1	1	0	0	0	Low
[38]	0	1	1	1	0	0	0	Low
[31]	0	1	1	1	0	0	0	Low
[29]	0	1	1	1	0	0	0	Low

[41]	0	1	1	1	0	0	0	Low
[39]	0	1	1	1	0	0	0	Low
[42]	0	1	1	1	0	0	0	Low
[30]	0	1	1	1	0	0	0	Low
[40]	0	1	1	1	0	0	0	Low
[2]	0	1	1	1	0	0	0	Low
[37]	0	1	1	1	0	0	0	Low
[44]	0	1	1	1	0	0	2	Moderate

3 Impact of Low Protein Diets on Growth Performance and Intestinal Morphology

3.1 Main Meta-Analysis Results (Table S3.1.1)

Table S3.1.1 Meta-analysis of the impact of low protein diets on the growth performance and intestinal morphology of weaned pigs

Outcomes	Pooled estimate (95% CI)	P	$P_{heterogeneity}$	I^2	Egger's test	Meta-reg (CP level)	Meta-reg (weaned ages)	Meta-reg (duration)	Meta-reg (initial BW)
ADG	SMD -1.322 (-1.728 to -0.916)	0.0001	0.0001	85.9%	0.0001	0.134	0.457	0.768	0.759
ADFI	SMD -0.457 (-1.007 to 0.093)	0.103	0.0001	88.0%	0.180	0.162	0.619	0.279	0.608
F:G ratio	SMD 1.136 (0.338 to 1.935)	0.005	0.0001	86.6%	0.148	-	-		
G:F ratio	SMD -1.584 (-2.233 to -0.936)	0.0001	0.0001	78.0%	0.132	-	-		
Final BW	SMD -1.003 (-1.384 to -0.622)	0.0001	0.0001	82.3%	0.001	-	-	0.506	0.068
Fecal consistency	SMD -1.538 (-2.106 to -0.970)	0.0001	0.225	28.0%	0.0001	-	-		
Duodenum VH	SMD -0.504 (-1.569 to 0.560)	0.353	0.0001	80.7%	0.773	-	-		
Duodenum CD	SMD -0.598 (-1.227 to 0.031)	0.063	0.075	50.1%	0.016	-	-		
Duodenum V:C	SMD 0.245 (-0.815 to 1.306)	0.650	0.001	77.3%	0.503	-	-		
Jejunum VH	SMD -1.152 (-1.612 to -0.691)	0.0001	0.666	0.0%	0.837	-	-		
Jejunum CD	SMD -0.716 (-1.230 to -0.202)	0.006	0.244	25.4%	0.033	-	-		
Jejunum V:C	SMD -0.023 (-0.500 to 0.454)	0.925	0.286	19.5%	0.277	-	-		

Ileum VH	SMD -0.504 (-1.569 to 0.560)	0.353	0.0001	80.7%	0.163	-	-
Ileum CD	SMD -0.598 (-1.227 to 0.031)	0.063	0.075	50.1%	0.0001	-	-
Ileum V:C	SMD 0.245 (-0.815 to 1.306)	0.650	0.001	77.3%	0.426	-	-

-- indicates no value

3.2 Sensitivity Analysis (Table S3.2.1 - 3.2.15)

Table S3.2.1 Sensitivity analysis for ADG: Pooled SMD and 95% CI after omitting each study

Study omitted	Estimate	95% Conf. Interval
[3]	-1.298	[-1.711, -0.885]
[33]	-1.316	[-1.729, -0.902]
[36]	-1.327	[-1.750, -0.903]
[38]	-1.387	[-1.807, -0.967]
[38]	-1.301	[-1.715, -0.887]
[35]	-1.322	[-1.740, -0.904]
[5]	-1.224	[-1.613, -0.835]
[34]	-1.283	[-1.692, -0.875]
[6]	-1.276	[-1.683, -0.869]
[6]	-1.321	[-1.736, -0.906]
[6]	-1.283	[-1.692, -0.875]
[28]	-1.298	[-1.710, -0.886]
[32]	-1.321	[-1.736, -0.907]
[32]	-1.321	[-1.736, -0.906]
[43]	-1.283	[-1.692, -0.875]

[31]	-1.370	[-1.786, -0.955]
[29]	-1.295	[-1.706, -0.883]
[41]	-1.317	[-1.736, -0.899]
[39]	-1.298	[-1.710, -0.886]
[42]	-1.327	[-1.743, -0.910]
[42]	-1.327	[-1.743, -0.910]
[42]	-1.327	[-1.743, -0.910]
[30]	-1.399	[-1.809, -0.989]
[2]	-1.385	[-1.803, -0.967]
[37]	-1.378	[-1.832, -0.925]
[37]	-1.382	[-1.744, -1.020]
[37]	-1.350	[-1.781, -0.920]
[40]	-1.283	[-1.692, -0.875]
Combined	-1.322	[-1.728, -0.916]

Table 3.2.1 Sensitivity analysis for ADFI: Pooled SMD and 95% CI after omitting each study

Study omitted	Estimate	[95% Conf. Interval]
[3]	-0.403	[-0.961, 0.155]
[33]	-0.429	[-0.993, 0.135]
[36]	-0.459	[-1.055, 0.138]
[38]	-0.547	[-1.087, -0.008]
[38]	-0.570	[-1.084, -0.056]
[35]	-0.415	[-0.979, 0.150]
[5]	-0.539	[-1.093, 0.014]
[34]	-0.379	[-0.932, 0.173]
[6]	-0.379	[-0.932, 0.173]
[6]	-0.376	[-0.927, 0.176]
[6]	-0.427	[-0.992, 0.137]
[28]	-0.429	[-0.996, 0.137]
[32]	-0.516	[-1.079, 0.048]
[32]	-0.516	[-1.079, 0.048]
[43]	-0.379	[-0.932, 0.173]
[31]	-0.484	[-1.053, 0.085]

[29]	-0.394	[-0.951, 0.162]
[41]	-0.445	[-1.024, 0.134]
[39]	-0.450	[-1.021, 0.121]
[42]	-0.444	[-1.014, 0.126]
[42]	-0.444	[-1.014, 0.126]
[42]	-0.444	[-1.014, 0.126]
[30]	-0.526	[-1.088, 0.036]
[2]	-0.473	[-1.056, 0.109]
[40]	-0.562	[-1.110, -0.015]
Combined	-0.457	[-1.007, 0.093]

Table 3.2.2 Sensitivity analysis for F:G ratio: Pooled SMD and 95% CI after omitting each study

Study omitted	Estimate	95% Conf. Interval
[3]	1.060	[0.151, 1.969]
[38]	0.965	[0.149, 1.782]
[38]	1.085	[0.163, 2.008]
[5]	0.917	[0.141, 1.692]
[32]	1.385	[0.648, 2.121]
[32]	1.390	[0.657, 2.122]
[43]	1.024	[0.188, 1.860]
[31]	1.242	[0.412, 2.073]
[42]	1.130	[0.261, 1.998]
[42]	1.131	[0.262, 2.000]
[42]	1.128	[0.259, 1.997]
[40]	1.172	[0.316, 2.028]
Combined	1.136	[0.338, 1.935]

Table 3.2.3 Sensitivity analysis for G:F ratio: Pooled SMD and 95% CI after omitting each study

Study omitted	Estimate	95% Conf. Interval
[3]	-1.578	[-2.267, -0.888]
[36]	-1.533	[-2.232, -0.834]
[35]	-1.708	[-2.409, -1.008]
[34]	-1.646	[-2.347, -0.945]
[6]	-1.485	[-2.145, -0.825]
[6]	-1.556	[-2.243, -0.868]
[6]	-1.454	[-2.096, -0.811]
[28]	-1.541	[-2.230, -0.853]
[29]	-1.553	[-2.246, -0.861]
[41]	-1.655	[-2.397, -0.912]
[39]	-1.553	[-2.246, -0.861]
[30]	-1.584	[-2.233, -0.936]
[2]	-1.741	[-2.222, -1.259]
Combined	-1.584	[-2.233, -0.936]

Table 3.2.4 Sensitivity analysis for final BW: Pooled SMD and 95% CI after omitting each study

Study omitted	Estimate	95% Conf. Interval
[3]	-0.950	[-1.329, -0.571]
[33]	-0.986	[-1.374, -0.598]
[36]	-0.986	[-1.380, -0.593]
[38]	-1.072	[-1.468, -0.676]
[38]	-1.010	[-1.410, -0.609]
[34]	-0.951	[-1.331, -0.571]
[6]	-0.951	[-1.331, -0.571]
[6]	-0.949	[-1.328, -0.569]
[6]	-0.988	[-1.377, -0.599]
[28]	-0.983	[-1.373, -0.594]
[32]	-1.031	[-1.424, -0.638]
[32]	-1.031	[-1.424, -0.638]
[29]	-0.960	[-1.343, -0.576]
[41]	-0.978	[-1.368, -0.588]
[39]	-0.991	[-1.381, -0.600]
[42]	-1.000	[-1.393, -0.608]

[42]	-1.000	[-1.392, -0.608]
[42]	-1.000	[-1.392, -0.607]
[30]	-1.081	[-1.467, -0.695]
[2]	-1.003	[-1.384, -0.622]
[37]	-1.063	[-1.494, -0.633]
[37]	-1.067	[-1.403, -0.732]
[37]	-1.032	[-1.439, -0.624]
[40]	-1.017	[-1.410, -0.625]
Combined	-1.003	[-1.384, -0.622]

Table 3.2.5 Sensitivity analysis for faecal consistency: Pooled SMD and 95% CI after omitting each study

Study omitted	Estimate	95% Conf. Interval
[38]	-1.353	[-1.850, -0.855]
[38]	-1.344	[-1.831, -0.857]
[35]	-1.728	[-2.358, -1.098]
[42]	-1.633	[-2.346, -0.920]
[42]	-1.628	[-2.341, -0.915]
[42]	-1.637	[-2.349, -0.925]
Combined	-1.538	[-2.106, -0.970]

Table 3.2.6 Sensitivity analysis for VH in the duodenum: Pooled SMD and 95% CI after omitting each study

Study omitted	Estimate	95% Conf. Interval
[38]	-0.779	[-1.903, 0.345]
[43]	-0.354	[-1.593, 0.885]
[29]	-0.913	[-1.770, -0.057]
[42]	-0.328	[-1.570, 0.914]
[42]	-0.328	[-1.570, 0.914]
[42]	-0.328	[-1.570, 0.914]
Combined	-0.504	[-1.569, 0.560]

Table 3.2.7 Sensitivity analysis for CD in the duodenum: Pooled SMD and 95% CI after omitting each study

Study omitted	Estimate	95% Conf. Interval
[38]	-0.498	[-1.199, 0.202]
[43]	-0.620	[-1.382, 0.142]
[29]	-0.309	[-0.781, 0.163]
[42]	-0.705	[-1.469, 0.060]
[42]	-0.772	[-1.452, -0.092]
[42]	-0.705	[-1.469, 0.060]
Combined	-0.598	[-1.227, 0.031]

Table 3.2.8 Sensitivity analysis for V:C ratio in the duodenum: Pooled SMD and 95% CI after omitting each study

Study omitted	Estimate	95% Conf. Interval
[38]	0.050	[-1.196, 1.296]
[43]	0.590	[-0.505, 1.684]
[29]	-0.192	[-1.026, 0.642]
[42]	0.392	[-0.984, 1.768]
[42]	0.392	[-0.984, 1.768]
[42]	0.245	[-0.815, 1.306]
Combined	0.245	[-0.815, 1.306]

Table 3.2.9 Sensitivity analysis for VH in the jejunum: Pooled SMD and 95% CI after omitting each study

Study omitted	Estimate	95% Conf. Interval
[38]	-1.339	[-1.843, -0.834]
[43]	-1.131	[-1.626, -0.636]
[29]	-1.113	[-1.620, -0.605]
[42]	-1.109	[-1.616, -0.601]
[42]	-1.109	[-1.616, -0.601]
[42]	-1.109	[-1.616, -0.601]
Combined	-1.152	[-1.612, -0.691]

Table 3.2.10 Sensitivity analysis for CD in the jejunum: Pooled SMD and 95% CI after omitting each study

Study omitted	Estimate	95% Conf. Interval
[38]	-0.659	[-1.250, -0.069]
[43]	-0.760	[-1.381, -0.139]
[29]	-0.492	[-0.964, -0.019]
[42]	-0.816	[-1.429, -0.202]
[42]	-0.816	[-1.429, -0.202]
[42]	-0.816	[-1.429, -0.202]
Combined	-0.716	[-1.230, -0.202]

Table 3.2.11 Sensitivity analysis for V:C ratio in the jejunum: Pooled SMD and 95% CI after omitting each study

Study omitted	Estimate	95% Conf. Interval
[38]	-0.063	[-0.635, 0.509]
[43]	0.142	[-0.312, 0.596]
[29]	-0.192	[-0.661, 0.277]
[42]	-0.021	[-0.615, 0.572]
[42]	-0.021	[-0.615, 0.572]
[42]	-0.022	[-0.615, 0.572]
Combined	-0.023	[-0.500, 0.454]

Table 3.2.12 Sensitivity analysis for VH in the ileum: Pooled SMD and 95% CI after omitting each study

Study omitted	Estimate	95% Conf. Interval
[38]	-0.736	[-1.315, -0.157]
[43]	-0.583	[-1.219, 0.052]
[29]	-0.380	[-0.849, 0.089]
[42]	-0.674	[-1.333, -0.015]
[42]	-0.674	[-1.333, -0.015]
[42]	-0.674	[-1.333, -0.015]
Combined	-0.612	[-1.147, -0.077]

Table 3.2.13 Sensitivity analysis for CD in the ileum: Pooled SMD and 95% CI after omitting each study

Study omitted	Estimate	95% Conf. Interval
[38]	-0.820	[-1.294, -0.346]
[43]	-0.830	[-1.305, -0.355]
[29]	-0.949	[-1.447, -0.451]
[42]	-0.974	[-1.473, -0.475]
[42]	-0.974	[-1.473, -0.475]
[42]	-0.974	[-1.473, -0.475]
Combined	-0.917	[-1.364, -0.469]

Table 3.2.14 Sensitivity analysis for V:C ratio in the ileum: Pooled SMD and 95% CI after omitting each study

Study omitted	Estimate	95% Conf. Interval
[38]	-0.207	[-1.062, 0.648]
[43]	0.294	[-0.238, 0.827]
[29]	-0.004	[-0.774, 0.765]
[42]	-0.150	[-1.113, 0.813]
[42]	0.101	[-0.859, 1.061]
[42]	-0.125	[-1.111, 0.861]
Combined	-0.004	[-0.774, 0.765]

3.3 Subgroup Analyses by CP Level, Weaning Age, treatment duration and initial BW (Table S3.3.1)

Table S3.3.1. Subgroup analyses of the impact of low protein diets on the growth performance and intestinal morphology of weaned pigs

Outcomes	Pooled estimate (95% CI)	P	$P_{heterogeneity}$	I^2	Subgroup	Subgroup No.
ADG overall	SMD -1.322 (-1.728 to -0.916)	0.0001	0.0001	85.9%		
16% subgroup	SMD -1.652 (-2.124 to -1.179)	0.0001	0.770	0.0%	CP level	4
17% subgroup	SMD -1.146 (-1.622 to -0.670)	0.0001	0.0001	85.5%	CP level	5
13% subgroup	SMD -2.502 (-4.681 to -0.324)	0.024	0.009	85.3%	CP level	1
14% subgroup	SMD -2.049 (-2.926 to -1.173)	0.0001	0.911	0.0%	CP level	2
15% subgroup	SMD -0.806 (-2.258 to 0.647)	0.277	0.001	86.4%	CP level	3
> 21d subgroup	SMD -1.343 (-2.044 to -0.642)	0.0001	0.0001	92.1%	Weaned ages	3
= 21d subgroup	SMD -1.406 (-1.843 to -0.970)	0.0001	0.251	22.4%	Weaned ages	2
< 21d subgroup	SMD -1.411 (-2.136 to -0.687)	0.0001	0.0001	75.4%	Weaned ages	1
< 4 weeks	SMD -0.950 (-1.564 to -0.336)	0.0001	0.002	89.9%	treatment duration	< 4 weeks
≥ 4 weeks	SMD -1.124 (-1.782 to -0.539)	0.0001	0.001	81.2%	treatment duration	≥ 4 weeks
< 8 kg	SMD -0.948 (-1.752 to -0.145)	0.0001	0.021	88.1%	initial BW	< 8 kg
≥ 8 kg	SMD -1.063 (-1.671 to -0.455)	0.0001	0.001	89.5%	initial BW	≥ 8 kg
ADFI overall	SMD -0.457 (-1.007 to 0.093)	0.103	0.0001	88.0%		

16% subgroup	SMD -1.203 (-1.758 to -0.648)	0.0001	0.245	29.0%	CP level	4
17% subgroup	SMD -0.658 (-1.355 to 0.039)	0.064	0.0001	88.1%	CP level	5
13% subgroup	SMD 1.261 (0.636 to 1.885)	0.0001	0.597	0.0%	CP level	1
14% subgroup	SMD -1.515 (-2.348 to -0.683)	0.0001	0.302	6.2%	CP level	2
15% subgroup	SMD 1.737 (0.269 to 3.205)	0.020	0.111	60.6%	CP level	3
> 21 subgroup	SMD -0.476 (-1.605 to 0.654)	0.409	0.0001	91.2%	Weaned ages	3
= 21 subgroup	SMD -0.436 (-1.068 to 0.195)	0.175	0.004	66.8%	Weaned ages	2
< 21 subgroup	SMD -0.566 (-1.815 to 0.682)	0.374	0.0001	91.8%	Weaned ages	1
< 4 weeks	SMD -0.092 (-0.853 to 0.668)	0.0001	0.812	84.3%	treatment duration	< 4 weeks
≥ 4 weeks	SMD -0.743 (-1.315 to -0.172)	0.0001	0.011	77.1%	treatment duration	≥ 4 weeks
< 8 kg	SMD -0.228 (-0.918 to 0.462)	0.0001	0.517	85.9%	initial BW	< 8 kg
≥ 8 kg	SMD -0.555 (-1.429 to 0.319)	0.0001	0.213	85.1%	initial BW	≥ 8 kg
Final BW	SMD -1.003 (-1.384 to -0.622)	0.0001	0.0001	82.3%		
< 4 weeks	SMD -0.933 (-1.411 to -0.455)	0.0001	0.0001	81.5%	treatment duration	< 4 weeks
≥ 4 weeks	SMD -1.213 (-1.912 to -0.514)	0.0001	0.001	77.5%	treatment duration	≥ 4 weeks
< 8 kg	SMD -1.321 (-1.645 to -0.998)	0.658	0.0001	0.0%	initial BW	< 8 kg
≥ 8 kg	SMD -0.730 (--1.304 to -0.157)	0.0001	0.013	88.6%	initial BW	≥ 8 kg

3.4 Effects of low-protein diets on growth performance of weaned piglets: subgroup analyses by treatment duration and initial body weight (Figure S2)

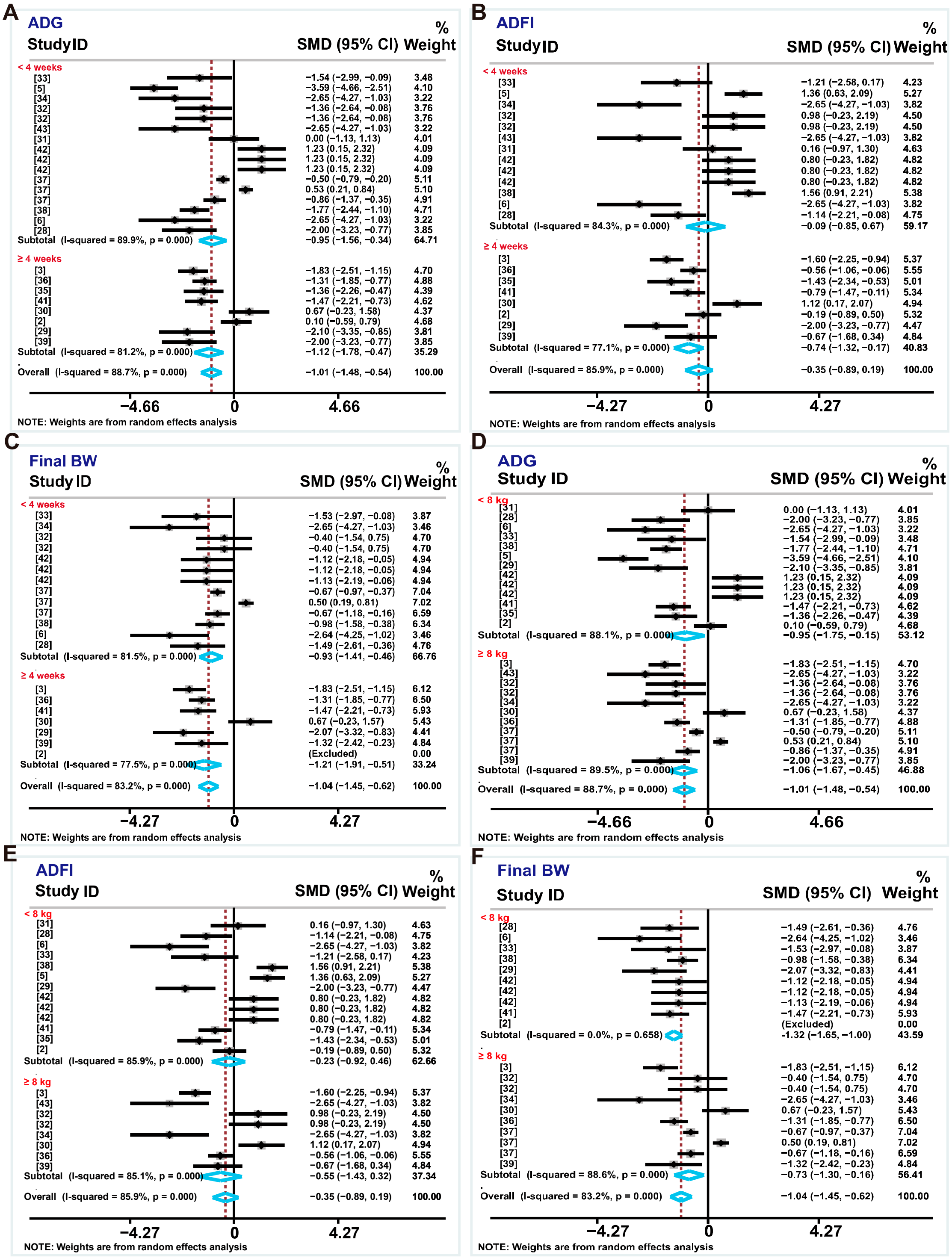

Figure S2. Subgroup analysis of different treatment duration and initial BW forest plot of the effects of low-protein diets on growth performance in weaned pigs. Subgroup analysis of ADG (A) , ADFI (B) and Final BW (C) in weaned pigs (subgroup with treatment duration < 4 weeks ; subgroup with treatment duration ≥ 4 weeks); Subgroup analysis of ADG (D), ADFI (E) and Final BW (F) in weaned pigs (subgroup < 8 kg with initial body weight < 8 kg; subgroup

< 8 kg with initial body weight < 8 kg). Control group = normal protein diet; Experimental group = low-protein diet; SMD = standard mean difference; 95%CI = 95% confidence interval; I^2 (0~100%) = test for heterogeneity; Test for overall effect *p* value (significance level $p < 0.05$). Diamonds on the positive quadrant of the X-axis favor an increase in the growth parameters, whereas those on the negative quadrant favor a decrease.

3.5 Trim-and-fill funnel plot for the effects of low-protein diets on ADG and ADFI in weaned piglets.

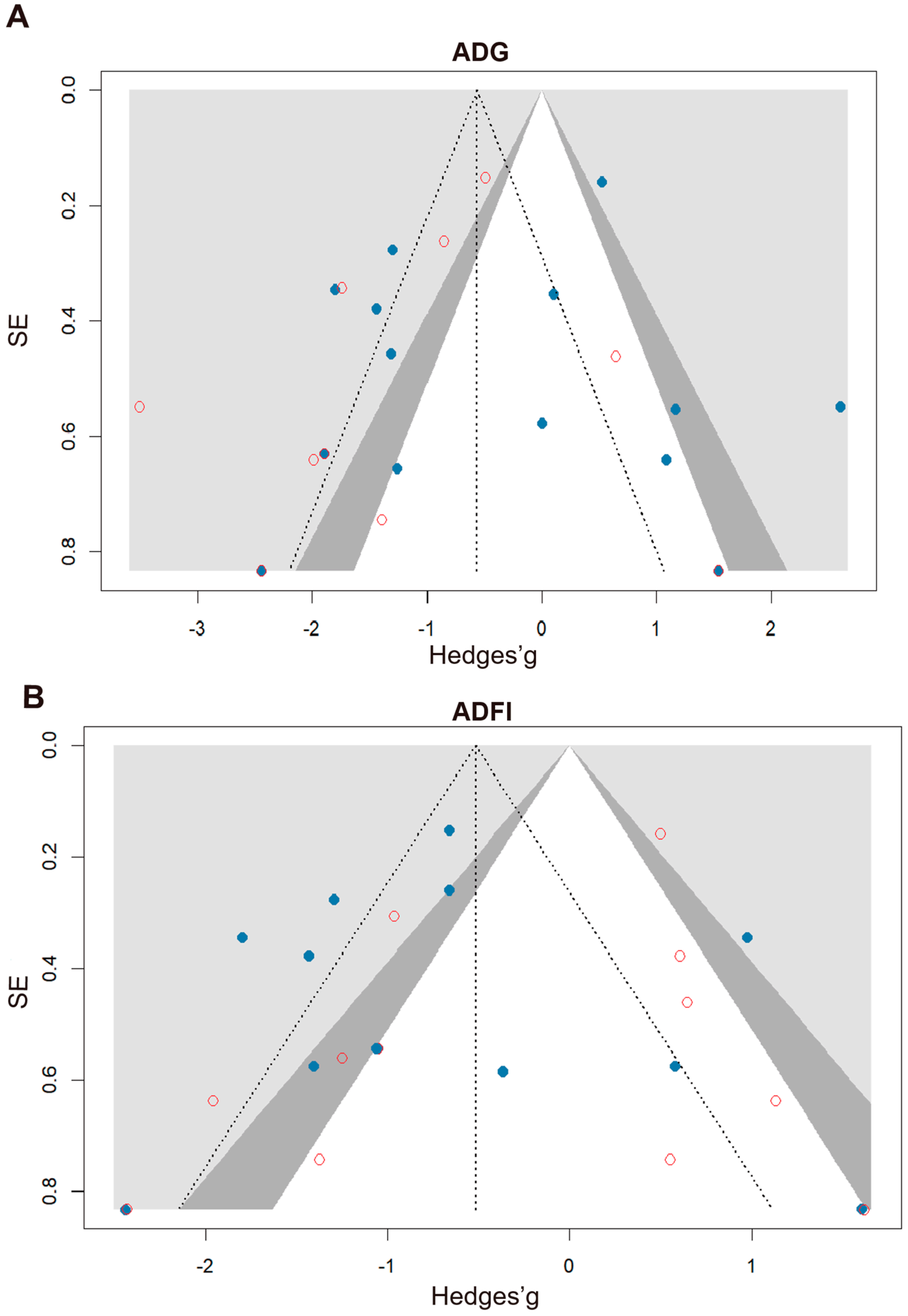

Figure S3. Trim-and-fill funnel plot for the effects of low-protein diets on ADG and ADFI in weaned piglets. Trim-and-fill funnel plot of the effect of a low-protein diets on ADG (A), ADFI (B)

4 Impact of Feed Additives on Growth Performance and Intestinal Morphology

4.1 Main Meta-Analysis Results (Table S4.1.1)

Table S4.1.1 Meta-analysis was conducted to investigate the impact of feed additives on the growth performance and intestinal morphology of weaned pigs.

Outcomes	Pooled estimate (95% CI)	P	$P_{heterogeneity}$	I^2	Egger's test	Meta-reg (CP level)	Meta-reg (Weaned ages)	Meta-reg (Type)	Meta-reg (duration)	Meta-reg (initial BW)
ADG	SMD 0.216 (-0.436 to 0.868)	0.516	0.0001	91.8%	0.046	0.257	0.119	0.033	0.138	0.033
ADFI	SMD 0.740 (0.332 to 1.147)	0.0001	0.0001	82.2%	0.279	0.875	0.536	0.809	0.048	0.809
F:G ratio	SMD 0.645 (-0.376 to 1.666)	0.216	0.0001	87.5%	0.0001	-	-	-	-	-
G:F ratio	SMD -0.088 (-0.670 to 0.494)	0.766	0.0001	86.5%	0.303	-	-	-	0.709	0.732
Final BW	SMD 0.240 (-0.423 to 0.903)	0.478	0.0001	91.5%	0.026	-	-	-	0.006	0.078
Fecal	SMD 1.313 (0.747 to	0.0001	0.266	24.3%	0.011	-	-	-	-	-

consistency	1.879)									
Duodenum VH	SMD 0.806 (-0.155 to 1.767)	0.100	0.001	74.9%	0.544	-	-	-	-	-
Duodenum CD	SMD 0.698 (-0.556 to 1.953)	0.275	0.0001	84.2%	0.572	-	-	-	-	-
Duodenum V:C	SMD -0.608 (-2.058 to 0.841)	0.411	0.0001	87.4%	0.661	-	-	-	-	-
Jejunum VH	SMD 0.610 (-0.513 to 1.734)	0.287	0.0001	81.2%	0.360	-	-	-	-	-
Jejunum CD	SMD 1.078 (-0.078 to 2.234)	0.067	0.0001	80.4%	0.852	-	-	-	-	-
Jejunum V:C	SMD -0.452 (-1.919 to 1.014)	0.545	0.010	78.1%	0.997	-	-	-	-	-
Ileum VH	SMD 1.635 (0.029 to 3.241)	0.046	0.0001	87.2%	0.289	-	-	-	-	-
Ileum CD	SMD 0.789 (0.315 to 1.264)	0.001	0.365	8.0%	0.0001	-	-	-	-	-

Ileum V:C	SMD 0.595 (-0.212 to 1.403)	0.149	0.040	60.0%	0.247	-	-	-	-	-

4.2 Sensitivity Analysis (Table 4.2.1-4.2.15)

Table 4.2.1 Sensitivity analysis for ADG: Pooled SMD and 95% CI after omitting each study

Study omitted	Estimate	95% Conf. Interval
[3]	0.302	[-0.356, 0.961]
[3]	0.302	[-0.356, 0.961]
[3]	0.302	[-0.356, 0.961]
[36]	0.278	[-0.392, 0.947]
[36]	0.278	[-0.392, 0.947]
[35]	0.170	[-0.499, 0.839]
[35]	0.170	[-0.499, 0.839]
[35]	0.170	[-0.499, 0.839]
[34]	0.130	[-0.527, 0.788]
[34]	0.130	[-0.527, 0.788]
[28]	0.147	[-0.514, 0.808]
[28]	0.216	[-0.436, 0.868]
[28]	0.135	[-0.523, 0.793]
[43]	0.315	[-0.343, 0.973]
[31]	0.226	[-0.448, 0.900]

[29]	0.138	[-0.521, 0.797]
[29]	0.312	[-0.347, 0.971]
[29]	0.142	[-0.518, 0.802]
[41]	0.164	[-0.504, 0.831]
[41]	0.288	[-0.382, 0.958]
[41]	0.164	[-0.504, 0.831]
[41]	0.288	[-0.382, 0.958]
[39]	0.220	[-0.457, 0.897]
[39]	0.233	[-0.444, 0.911]
[30]	0.290	[-0.376, 0.957]
[2]	0.179	[-0.496, 0.853]
[2]	0.179	[-0.496, 0.853]
[40]	0.191	[-0.441, 0.824]
Combined	0.216	[-0.436, 0.868]

Table 4.2.2 Sensitivity analysis for ADFI: Pooled SMD and 95% CI after omitting each study

Study omitted	Estimate	95% Conf. Interval
[3]	0.704	[0.287, 1.121]
[3]	0.704	[0.287, 1.121]
[3]	0.704	[0.287, 1.121]
[36]	0.788	[0.374, 1.201]
[36]	0.788	[0.374, 1.201]
[35]	0.714	[0.295, 1.133]
[35]	0.714	[0.295, 1.133]
[35]	0.714	[0.295, 1.133]
[34]	0.687	[0.279, 1.096]
[34]	0.687	[0.279, 1.096]
[28]	0.727	[0.308, 1.147]
[28]	0.724	[0.305, 1.143]
[28]	0.727	[0.307, 1.146]
[43]	0.687	[0.279, 1.096]
[31]	0.756	[0.337, 1.175]
[29]	0.828	[0.435, 1.220]

[29]	0.698	[0.285, 1.110]
[29]	0.698	[0.285, 1.110]
[41]	0.801	[0.399, 1.204]
[41]	0.741	[0.313, 1.168]
[41]	0.802	[0.399, 1.204]
[41]	0.802	[0.399, 1.204]
[39]	0.749	[0.328, 1.170]
[39]	0.750	[0.329, 1.172]
[30]	0.791	[0.379, 1.204]
[2]	0.747	[0.320, 1.174]
[2]	0.747	[0.320, 1.174]
[40]	0.742	[0.322, 1.161]
Combined	0.740	[0.332, 1.147]

Table 4.2.3 Sensitivity analysis for F:G ratio: Pooled SMD and 95% CI after omitting each study

Study omitted	Estimate	95% Conf. Interval
[3]	0.771	[-0.343, 1.886]
[3]	0.335	[-0.937, 1.606]
[3]	0.769	[-0.346, 1.885]
[43]	0.347	[-0.943, 1.636]
[31]	0.349	[-0.944, 1.642]
[40]	1.207	[0.467, 1.947]
Combined	0.645	[-0.376, 1.666]

Table 4.2.4 Sensitivity analysis for G:F ratio: Pooled SMD and 95% CI after omitting each study

Study omitted	Estimate	95% Conf. Interval
[36]	-0.200	[-0.776, 0.376]
[36]	0.012	[-0.575, 0.599]
[35]	-0.088	[-0.670, 0.494]
[35]	-0.071	[-0.691, 0.549]
[35]	-0.103	[-0.721, 0.514]
[34]	-0.168	[-0.758, 0.422]
[34]	-0.157	[-0.751, 0.438]
[28]	0.009	[-0.579, 0.598]
[28]	-0.088	[-0.670, 0.494]
[28]	-0.088	[-0.670, 0.494]
[29]	-0.208	[-0.780, 0.365]
[29]	0.009	[-0.579, 0.598]
[29]	-0.211	[-0.782, 0.360]
[41]	-0.017	[-0.623, 0.588]
[41]	-0.031	[-0.646, 0.584]
[41]	-0.031	[-0.646, 0.584]

[41]	-0.031	[-0.646, 0.584]
[39]	-0.091	[-0.703, 0.522]
[39]	-0.091	[-0.703, 0.522]
[30]	0.014	[-0.572, 0.600]
[2]	-0.154	[-0.751, 0.443]
[2]	-0.154	[-0.751, 0.443]
Combined	-0.088	[-0.670, 0.494]

Table 4.2.15 Sensitivity analysis for final BW: Pooled SMD and 95% CI after omitting each study

Study omitted	Estimate	95% Conf. Interval
[3]	0.339	[-0.332, 1.010]
[3]	0.339	[-0.332, 1.010]
[3]	0.339	[-0.332, 1.010]
[36]	0.311	[-0.372, 0.994]
[36]	0.310	[-0.373, 0.994]
[34]	0.144	[-0.523, 0.811]
[34]	0.144	[-0.523, 0.811]
[28]	0.184	[-0.494, 0.862]
[28]	0.184	[-0.494, 0.862]
[28]	0.182	[-0.495, 0.859]
[29]	0.159	[-0.511, 0.829]
[29]	0.340	[-0.335, 1.014]
[29]	0.159	[-0.511, 0.829]
[41]	0.180	[-0.495, 0.856]
[41]	0.320	[-0.366, 1.007]
[41]	0.180	[-0.496, 0.856]

[41]	0.325	[-0.359, 1.008]
[39]	0.262	[-0.430, 0.954]
[39]	0.245	[-0.447, 0.936]
[30]	0.322	[-0.360, 1.003]
[2]	0.205	[-0.484, 0.894]
[2]	0.207	[-0.483, 0.896]
[40]	0.144	[-0.523, 0.810]
Combined	0.240	[-0.423, 0.903]

Table 4.2.16 Sensitivity analysis for faecal consistency: Pooled SMD and 95% CI after omitting each study

Study omitted	Estimate	95% Conf. Interval
[38]	1.136	[0.635, 1.638]
[35]	1.503	[0.704, 2.301]
[35]	1.439	[0.578, 2.299]
[35]	1.439	[0.578, 2.299]
Combined	1.313	[0.747, 1.879]

Table 4.2.17 Sensitivity analysis for VH in the duodenum: Pooled SMD and 95% CI after omitting each study

Study omitted	Estimate	95% Conf. Interval
[44]	0.715	[-0.425, 1.855]
[44]	0.715	[-0.425, 1.855]
[43]	0.803	[-0.372, 1.978]
[29]	0.668	[-0.464, 1.799]
[29]	1.286	[0.754, 1.817]
[29]	0.668	[-0.464, 1.799]
Combined	0.806	[-0.155, 1.767]

Table 4.2.18 Sensitivity analysis for CD in the duodenum: Pooled SMD and 95% CI after omitting each study

Study omitted	Estimate	95% Conf. Interval
[44]	0.727	[-0.826, 2.280]
[44]	0.843	[-0.678, 2.363]
[43]	0.525	[-0.933, 1.983]
[29]	1.228	[0.386, 2.071]
[29]	0.425	[-0.957, 1.807]
[29]	0.425	[-0.957, 1.807]
Combined	0.698	[-0.556, 1.953]

Table 4.2.19 Sensitivity analysis for V:C ratio in the duodenum: Pooled SMD and 95% CI after omitting each study

Study omitted	Estimate	95% Conf. Interval
[44]	-0.486	[-2.237, 1.266]
[44]	-0.976	[-2.520, 0.569]
[43]	-0.414	[-2.113, 1.285]
[29]	-1.146	[-2.360, 0.068]
[29]	-0.315	[-1.942, 1.312]
[29]	-0.315	[-1.942, 1.312]
Combined	-0.608	[-2.058, 0.841]

Table 4.2.10 Sensitivity analysis for VH in the jejunum: Pooled SMD and 95% CI after omitting each study

Study omitted	Estimate	95% Conf. Interval
[44]	0.901	[-0.322, 2.124]
[44]	0.576	[-0.792, 1.944]
[43]	0.266	[-0.841, 1.373]
[29]	0.472	[-0.858, 1.801]
[29]	0.995	[-0.012, 2.003]
[29]	0.472	[-0.858, 1.801]
Combined	0.610	[-0.513, 1.734]

Table 4.2.11 Sensitivity analysis for CD in the jejunum: Pooled SMD and 95% CI after omitting each study

Study omitted	Estimate	95% Conf. Interval
[44]	1.579	[0.836, 2.322]
[44]	0.976	[-0.405, 2.358]
[43]	1.254	[-0.110, 2.618]
[29]	0.879	[-0.457, 2.214]
[29]	0.879	[-0.457, 2.214]
[29]	0.879	[-0.457, 2.214]
Combined	1.078	[-0.078, 2.234]

Table 4.2.12 Sensitivity analysis for V:C ratio in the jejunum: Pooled SMD and 95% CI after omitting each study

Study omitted	Estimate	95% Conf. Interval
[44]	-0.452	[-1.919, 1.014]
[44]	0.079	[-1.757, 1.915]
[43]	-1.115	[-1.927, -0.302]
[29]	-0.452	[-1.919, 1.014]
[29]	-0.261	[-2.850, 2.328]
[29]	-0.452	[-1.919, 1.014]
Combined	-0.452	[-1.919, 1.014]

Table 4.2.13 Sensitivity analysis for VH in the ileum: Pooled SMD and 95% CI after omitting each study

Study omitted	Estimate	95% Conf. Interval
[44]	1.444	[-0.410, 3.299]
[44]	1.444	[-0.410, 3.299]
[43]	1.444	[-0.410, 3.299]
[29]	1.554	[-0.434, 3.541]
[29]	2.341	[1.700, 2.982]
[29]	1.554	[-0.434, 3.541]
Combined	1.635	[0.029, 3.241]

Table 4.2.14 Sensitivity analysis for CD in the ileum: Pooled SMD and 95% CI after omitting each study

Study omitted	Estimate	95% Conf. Interval
[44]	0.897	[0.381, 1.413]
[44]	0.897	[0.381, 1.413]
[43]	0.897	[0.381, 1.413]
[29]	0.680	[0.157, 1.204]
[29]	0.680	[0.157, 1.204]
[29]	0.680	[0.157, 1.204]
Combined	0.789	[0.315, 1.264]

Table 4.2.15 Sensitivity analysis for V:C ratio in the ileum: Pooled SMD and 95% CI after omitting each study

Study omitted	Estimate	95% Conf. Interval
[44]	0.495	[-0.490, 1.480]
[44]	0.495	[-0.490, 1.480]
[43]	0.495	[-0.490, 1.480]
[29]	0.595	[-0.212, 1.403]
[29]	1.003	[0.421, 1.586]
[29]	0.520	[-0.515, 1.555]
Combined	0.595	[-0.212, 1.403]

4.3 Subgroup Analyses by Additive Type, CP Level, Weaning Age, treatment duration and initial BW (Table 4.3.1)

Table S4.3.1 Subgroup analyses were conducted to investigate the impact of feed additives on the growth performance and intestinal morphology of weaned pigs.

Outcomes	Pooled estimate (95% CI)	P	$P_{heterogeneity}$	I^2	Subgroup	Subgroup No.
ADG overall	SMD 0.216 (-0.436 to 0.868)	0.516	0.0001	91.8%		
16% subgroup	SMD -0.898 (-1.831 to 0.035)	0.059	0.0001	92.4%	CP level	3
17% subgroup	SMD 0.835 (0.216 to 1.454)	0.008	0.0001	75.1%	CP level	4
14% subgroup	SMD 1.282 (-0.459 to 3.023)	0.149	0.0001	89.2%	CP level	1
15% subgroup	SMD 43.565 (-49.373 to 136.504)	0.358	0.0001	95.2%	CP level	2
> 21d subgroup	SMD -0.011 (-0.957 to 0.934)	0.981	0.0001	93.7%	Weaned ages	2
= 21d subgroup	SMD 0.306 (-0.636 to 1.355)	0.479	0.0001	86.6%	Weaned ages	1
Fatty acid subgroup	SMD -1.830 (-2.221 to -1.438)	0.0001	1.000	0.0%	Type of feed additives	2
Vitamin subgroup	SMD -1.312 (-2.406 to -0.218)	0.019	-	-	Type of feed additives	3
Enzyme subgroup	SMD 0.383 (-0.990 to 1.756)	0.585	0.0001	87.2%	Type of feed additives	4
Amino acid subgroup	SMD 0.860 (0.001 to 1.719)	0.050	0.0001	90.6%	Type of feed additives	5
Plant extract subgroup	SMD 0.004 (-0.588 to 0.595)	0.991	0.898	0.0%	Type of feed additives	1
Fermentable carbohydrates subgroup	SMD -1.614 (-2.637 to -0.592)	0.002	-	-	Type of feed additives	6
< 4 weeks	SMD 1.169 (-0.369 to 2.708)	0.0001	0.136	86.3%	treatment duration	< 4 weeks

≥ 4 weeks	SMD -0.073 (-0.757 to 0.612)	0.0001	0.835	92.1%	treatment duration	≥ 4 weeks
< 8 kg	SMD 0.835 (0.124 to 1.546)	0.0001	0.021	88.9%	initial BW	< 8 kg
≥ 8 kg	SMD -0.756 (-1.534 to 0.022)	0.0001	0.057	85.5%	initial BW	≥ 8 kg
ADFI overall	SMD 0.740 (0.332 to 1.147)	0.0001	0.0001	82.2%		
16% subgroup	SMD 0.252 (-0.516 to 1.019)	0.521	0.0001	90.2%	CP level	3
17% subgroup	SMD 1.150 (0.720 to 1.581)	0.0001	0.033	49.2%	CP level	4
14% subgroup	SMD 0.898 (-0.234 to 2.030)	0.120	0.0001	82.8%	CP level	1
15% subgroup	SMD -0.0001 (-1.294 to 1.293)	1.000	0.079	67.6%	CP level	2
> 21d subgroup	SMD 0.938 (0.329 to 1.547)	0.003	0.0001	87.2%	Weaned ages	2
= 21d subgroup	SMD 0.499 (-0.169 to 1.167)	0.143	0.0001	75.8%	Weaned ages	1
Fatty acid subgroup	SMD 1.593 (1.216 to 1.970)	0.0001	1.000	0.0%	Type of feed additives	2
Vitamin subgroup	SMD -0.562 (-1.564 to 0.440)	0.272	-	-	Type of feed additives	3
Enzyme subgroup	SMD 0.320 (-0.361 to 1.001)	0.357	0.111	54.4%	Type of feed additives	4
Amino acid subgroup	SMD 0.913 (0.263 to 1.563)	0.006	0.0001	86.0%	Type of feed additives	5
Plant extract subgroup	SMD 0.459 (-0.141 to 1.059)	0.134	-	0.0%	Type of feed additives	1
Fermentable carbohydrates subgroup	SMD -0.606 (-1.505 to 0.292)	0.186	-	-	Type of feed additives	6
< 4 weeks	SMD 1.497 (0.846 to 2.148)	0.088	0.0001	45.6%	treatment duration	< 4 weeks

≥ 4 weeks	SMD 0.501 (0.021 to 0.982)	0.0001	0.041	85.3%	treatment duration	≥ 4 weeks
< 8 kg	SMD 0.575 (0.047 to 1.104)	0.0001	0.033	82.7%	initial BW	< 8 kg
≥ 8 kg	SMD 0.995 (0.328 to 1.663)	0.0001	0.003	81.5%	initial BW	≥ 8 kg
G: F Ratio Overall	SMD -0.088 (-0.670 to 0.494)	0.766	0.0001	86.5%		
< 4 weeks	SMD 0.243 (-1.995 to 2.480)	0.0001	0.832	89.4%	treatment duration	< 4 weeks
≥ 4 weeks	SMD -0.144 (-0.757 to 0.468)	0.0001	0.644	86.8%	treatment duration	≥ 4 weeks
< 8 kg	SMD -0.180 (-0.876 to 0.517)	0.0001	0.613	87.7%	initial BW	< 8 kg
≥ 8 kg	SMD 0.084 (-1.073 to 1.241)	0.0001	0.887	86.0%	initial BW	≥ 8 kg
Final BW Overall	SMD 0.240 (-0.423 to 0.903)	0.478	0.0001	91.5%		
< 4 weeks	SMD 1.784 (1.218 to 2.351)	0.575	0.0001	0.0%	treatment duration	< 4 weeks
≥ 4 weeks	SMD -0.336 (-1.047 to 0.375)	0.0001	0.354	91.6%	treatment duration	≥ 4 weeks
< 8 kg	SMD 0.699 (-0.115 to 1.514)	0.0001	0.092	89.8%	initial BW	< 8 kg
≥ 8 kg	SMD -0.591 (-1.395 to 0.213)	0.0001	0.150	86.1%	initial BW	≥ 8 kg

4.4 Effect of feed additives on growth performance of weaned piglets fed low-protein diets: subgroup analyses by treatment duration (Figure S4)

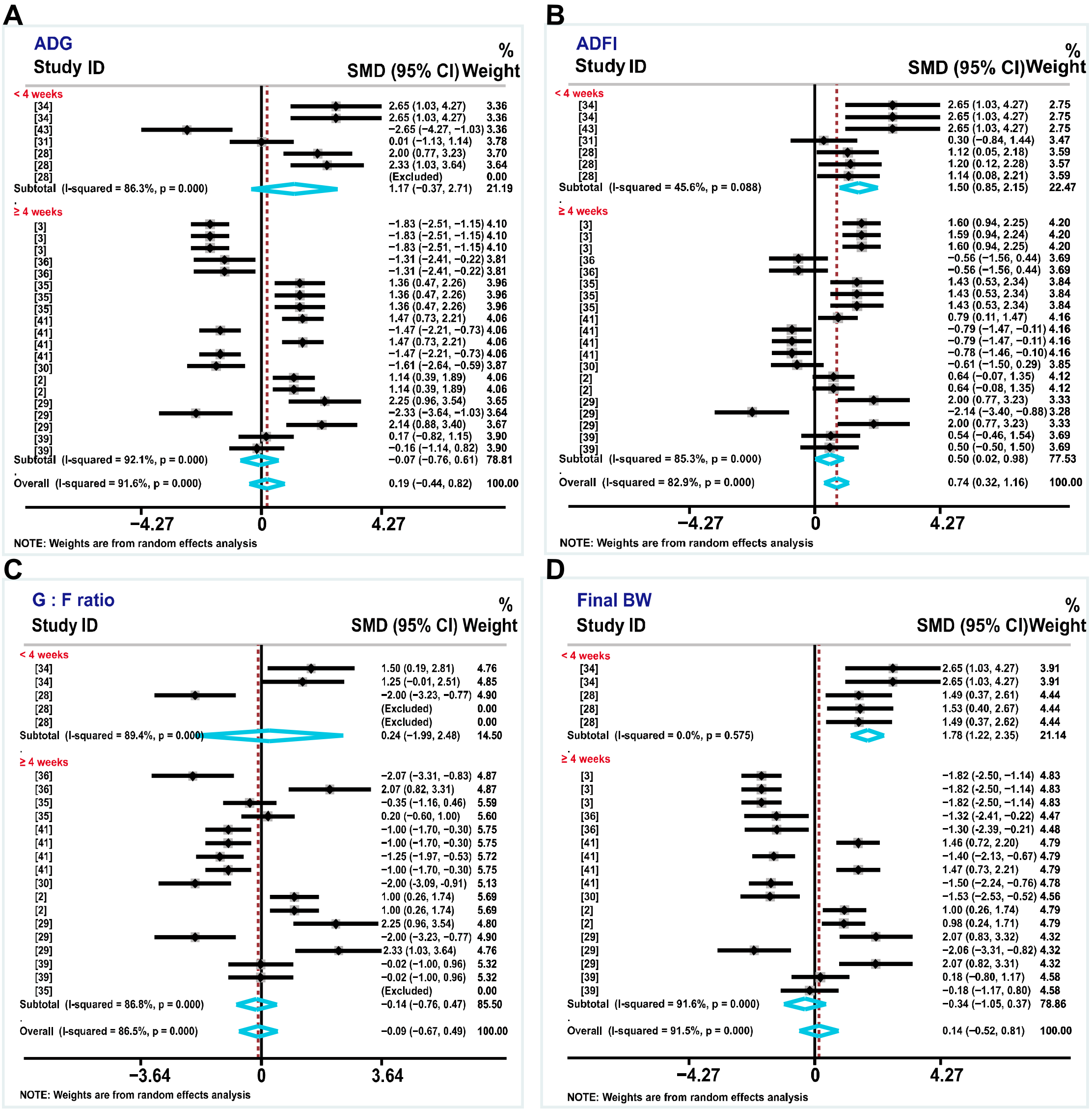

Figure S4. Effect of feed additives on growth performance of weaned piglets fed low-protein diets: subgroup analyses by treatment duration. Subgroup analysis of ADG (A) , ADFI (B) , G: F ratio (C) and Final BW (D) in weaned pigs (subgroup with treatment duration < 4 weeks ; subgroup with treatment duration ≥ 4 weeks); Subgroup analysis of ADG (D), ADFI (E) and Final BW (F) in weaned pigs (subgroup < 8 kg with initial body weight < 8 kg; subgroup < 8 kg with initial body weight < 8 kg). Control group = normal protein diet; Experimental group = low-protein diet; SMD = standard mean difference; 95%CI = 95% confidence interval; I^2 (0~100%) = test for heterogeneity; Test for overall effect *p* value (significance level *p* <0.05). Diamonds on the positive quadrant of the X-axis favor an increase in the growth parameters, whereas those on the negative quadrant favor a decrease.

4.5 Effect of feed additives on growth performance of weaned piglets fed low-protein diets: subgroup analyses by initial body weight (Figure S5)

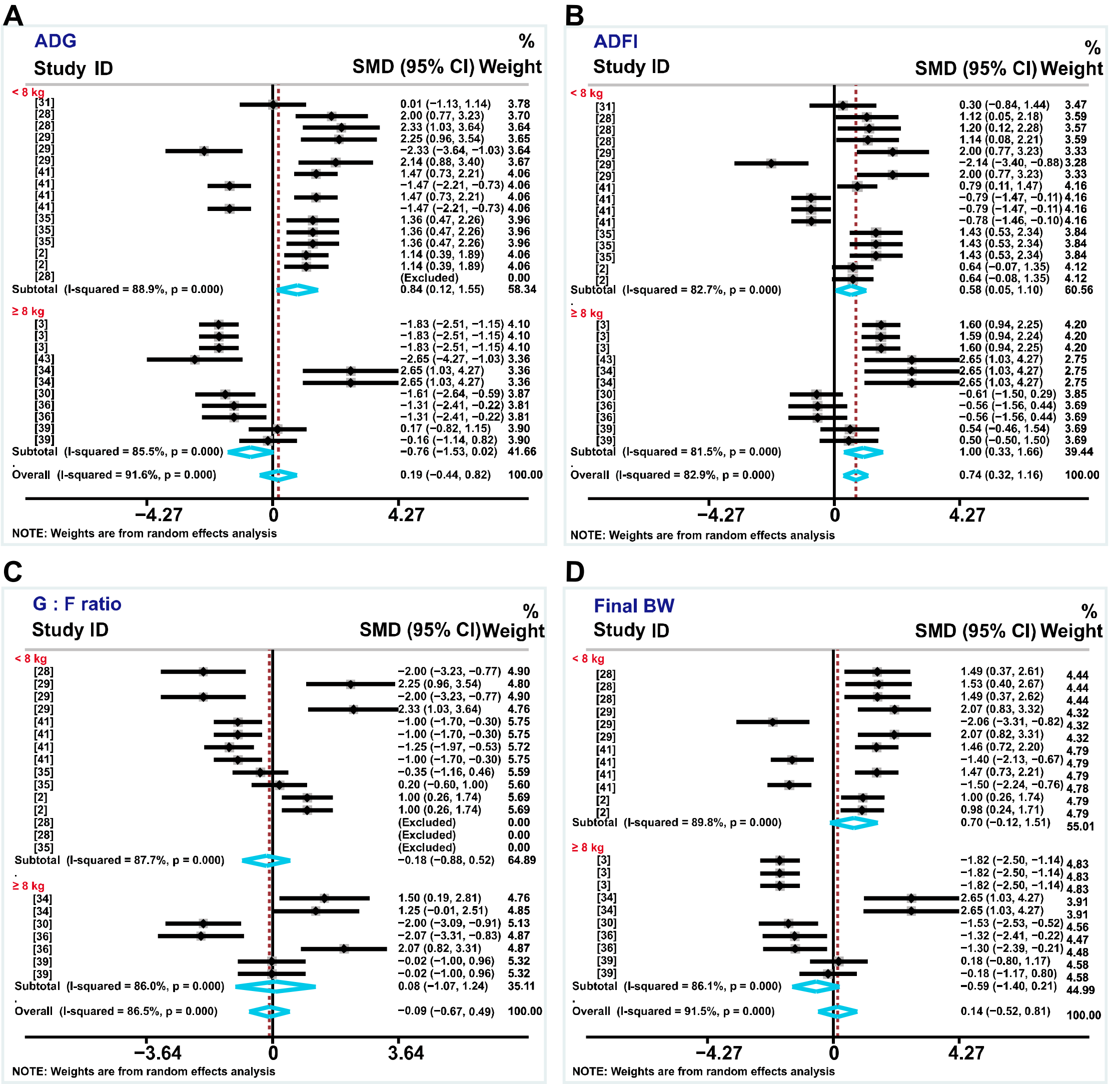

Figure S5. Effect of feed additives on growth performance of weaned piglets fed low-protein diets: subgroup analyses by initial body weight. Subgroup analysis of ADG (A), ADFI (B), G: F ratio (C) and Final BW (D) in weaned pigs (subgroup < 8 kg with initial body weight < 8 kg; subgroup < 8 kg with initial body weight < 8 kg). Control group = low-protein diet; Experimental group = low-protein diet; SMD = standard mean difference; 95%CI = 95% confidence interval; I^2 (0~100%) = test for heterogeneity; Test for overall effect p value (significance level p <0.05). Diamonds on the positive quadrant of the X-axis favor an increase in the growth parameters, whereas those on the negative quadrant favor a decrease.

4.6 Trim-and-fill funnel plot for the effect of feed additives on ADG and ADFI in weaned piglets fed low-protein diets

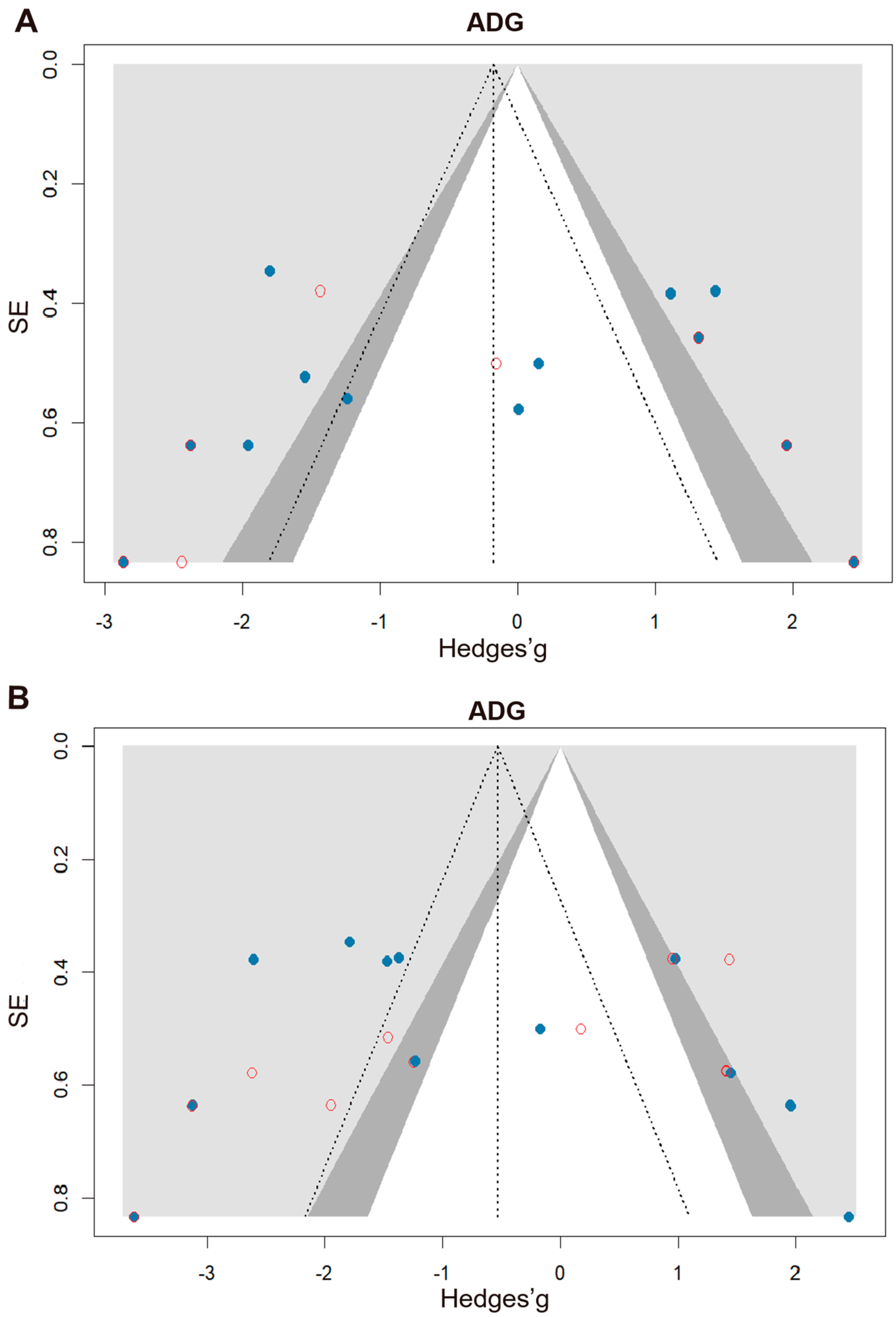

Figure S6. Trim-and-fill funnel plot for the effects of feed additives on ADG and ADFI in weaned piglets fed low-protein diets. Trim-and-fill funnel plot of the effect of feed additives on ADG (A), ADFI (B).

4.7 Efficacy Ranking of Nutritional Interventions (Table S4.4.1)

Table S4.4.1 Efficacy ranking of intervention measures

Item	Rank	Placebo	Plant extracts	Fatty acid	Vitamin	Enzymes	Amino acid	Fermentable carbohydrates
ADG	1	0.005	0.157	0.102	0.05	0.102	0.56	0.024
ADG	2	0.127	0.182	0.122	0.072	0.168	0.291	0.038
ADG	3	0.318	0.154	0.115	0.078	0.186	0.103	0.045
ADG	4	0.349	0.148	0.128	0.091	0.182	0.032	0.07
ADG	5	0.167	0.155	0.186	0.173	0.188	0.01	0.124
ADG	6	0.032	0.136	0.198	0.253	0.131	0.002	0.246
ADG	7	0.002	0.068	0.149	0.283	0.043	0.002	0.453
ADG	8	1	1	1	1	1	1	1
ADFI	1	0.002	0.149	0.243	0.064	0.052	0.429	0.062
ADFI	2	0.052	0.182	0.175	0.108	0.108	0.339	0.073
ADFI	3	0.225	0.168	0.142	0.145	0.145	0.158	0.08
ADFI	4	0.347	0.145	0.122	0.161	0.161	0.054	0.088
ADFI	5	0.273	0.145	0.121	0.205	0.205	0.015	0.125
ADFI	6	0.091	0.137	0.119	0.205	0.205	0.004	0.228
ADFI	7	0.01	0.074	0.078	0.124	0.124	0.001	0.344
ADFI	8	1	1	1	1	1	1	1
G: F ratio	1	0.048	0.47		0	0.262	0.18	0.049
G: F ratio	2	0.231	0.009		0	0.298	0.361	0.1
G: F ratio	3	0.406	0.005		0	0.184	0.282	0.118
G: F ratio	4	0.267	0.012		0	0.178	0.14	0.403
G: F ratio	5	0.048	0.504		0	0.078	0.037	0.33
G: F ratio	6	0.0001	0.0001		1	0	0	0
G: F ratio	7	1	1		1	1	1	1

References

2. Kim, Y.J.; Lee, J.H.; Kim, T.H.; Song, M.H.; Yun, W.; Oh, H.J.; Lee, J.S.; Kim, H.B.; Cho, J.H. Effect of low protein diets added with protease on growth performance, nutrient digestibility of weaned piglets and growing-finishing pigs. J. Anim. Sci. Technol. 2021, 63, 491–500.

3. Li, W.; Lan, T.; Ding, Q.; Ren, Z.; Tang, Z.; Tang, Q.; Peng, X.; Xu, Y.; Sun, Z. Effect of low protein diets supplemented with sodium butyrate, medium-chain fatty acids, or n-3 polyunsaturated fatty acids on the growth performance, immune function, and microbiome of weaned piglets. Int. J. Mol. Sci. 2023, 24, 17592.

5. Shi, Q.; Zhu, Y.; Wang, J.; Yang, H.; Wang, J.; Zhu, W. Protein restriction and succedent realimentation affecting ileal morphology, ileal microbial composition and metabolites in weaned piglets. Animal 2019, 13, 2463–2472.

6. Nyachoti, C.M.; Omogbenigun, F.O.; Rademacher, M.; Blank, G. Performance responses and indicators of gastrointestinal health in early-weaned pigs fed low-protein amino acid-supplemented diets. J. Anim. Sci. 2006, 84, 125–134.

28. Goodarzi, P.; Wileman, C.M.; Habibi, M.; Walsh, K.; Sutton, J.; Shili, C.N.; Chai, J.; Zhao, J.; Pezeshki, A. Effect of isoleucine and added valine on performance, nutrients digestibility and gut microbiota composition of pigs fed with very low protein diets. Int. J. Mol. Sci. 2022, 23, 14886.

29. Habibi, M.; Goodarzi, P.; Shili, C.N.; Sutton, J.; Wileman, C.M.; Kim, D.M.; Lin, D.; Pezeshki, A. A mixture of valine and isoleucine restores the growth of protein-restricted pigs likely through improved gut development, hepatic IGF-1 pathway, and plasma metabolomic profile. Int. J. Mol. Sci. 2022, 23, 3300.

30. Bikker, P.; Dirkzwager, A.; Fledderus, J.; Trevisi, P.; le Huerou-Luron, I.; Lalles, J.P.; Awati, A. Dietary protein and fermentable carbohydrates contents influence growth performance and intestinal characteristics in newly weaned pigs. Livest. Sci. 2007, 108, 194–197.

31. Cui, Z.; Wang, X.; Hou, Z.; Liao, S.; Qi, M.; Zha, A.; Yang, Z.; Zuo, G.; Liao, P.; Chen, Y.; et al. Low-protein diet supplemented with medium-chain fatty acid glycerides improves the growth performance and intestinal function in post-weaning piglets. Animals 2020, 10, 14.

32. Deng, D.; Yao, K.; Chu, W.; Li, T.; Huang, R.; Yin, Y.; Liu, Z.; Zhang, J.; Wu, G. Impaired translation initiation activation and reduced protein synthesis in weaned piglets fed a low-protein diet. J. Nutr. Biochem. 2009, 20, 544–552.

33. Hou, L.; Wang, L.; Qiu, Y.; Xiong, Y.; Xiao, H.; Yi, H.; Wen, X.; Lin, Z.; Wang, Z.; Yang, X.; et al. Effects of protein restriction and subsequent realimentation on body composition, gut microbiota and metabolite profiles in

weaned piglets. Animals 2021, 11, 17.

34. Li, Y.; Wei, H.; Li, F.; Duan, Y.; Guo, Q.; Yin, Y. Effects of low-protein diets supplemented with branched-chain amino acid on lipid metabolism in white adipose tissue of piglets. J. Agric. Food Chem. 2017, 65, 2839–2848.

35. Lordelo, M.M.; Gaspar, A.M.; Le Bellego, L.; Freire, J.P.B. Isoleucine and valine supplementation of a low-protein corn-wheat-soybean meal-based diet for piglets: Growth performance and nitrogen balance. J. Anim. Sci. 2008, 86, 2936–2941.

36. Ogunribido, T.Z.; Bedford, M.R.; Adeola, O.; Ajuwon, K.M. Effects of supplemental myo-inositol on growth performance and apparent total tract digestibility of weanling piglets fed reduced protein high-phytate diets and intestinal epithelial cell prolif-eration and function. J. Anim. Sci. 2022, 100, 10.

37. Lynegaard, J.C.; Kjeldsen, N.J.; Hansen, C.F.; Williams, A.R.; Nielsen, J.P.; Amdi, C. Reduction in diarrhoea and modulation of intestinal gene expression in pigs allocated a low protein diet without medicinal zinc oxide post-weaning. Animals 2022, 12, 15.

38. Opapeju, F.O.; Rademacher, M.; Blank, G.; Nyachoti, C.M. Effect of low-protein amino acid-supplemented diets on the growth performance, gut morphology, organ weights and digesta characteristics of weaned pigs. Animal 2008, 2, 1457–1464.

39. Shili, C.N.; Habibi, M.; Sutton, J.; Barnes, J.; Burch-Konda, J.; Pezeshki, A. Effect of a phytogenic water additive on growth performance, blood metabolites and gene expression of amino acid transporters in nursery pigs fed with low-protein/high-carbohydrate diets. Animals 2021, 11, 555.

40. Wan, K.; Li, Y.; Sun, W.; An, R.; Tang, Z.; Wu, L.; Chen, H.; Sun, Z. Effects of dietary calcium pyruvate on gastrointestinal tract development, intestinal health and growth performance of newly weaned piglets fed low-protein diets. J. Appl. Microbiol. 2020, 128, 355–365.

41. Wellington, M.O.; Hulshof, T.G.; Resink, J.W.; Ernst, K.; Balemans, A.; Page, G. The effect of supplementation of essential amino acid combinations in a low crude protein diet on growth performance in weanling pigs. Transl. Anim. Sci. 2023, 7, 7.

42. Yue, L.Y.; Qiao, S.Y. Effects of low-protein diets supplemented with crystalline amino acids on performance and intestinal de-velopment in piglets over the first 2 weeks after weaning. Livest. Sci. 2008, 115, 144–152.

43. Zhang, S.; Qiao, S.; Ren, M.; Zeng, X.; Ma, X.; Wu, Z.; Thacker, P.; Wu, G. Supplementation with branched-chain amino acids to a low-protein diet regulates intestinal expression of amino acid and peptide transporters in weanling pigs. Amino Acids 2013, 45, 1191–1205.

44. Zhou, J.; Wang, Y.; Zeng, X.; Zhang, T.; Li, P.; Yao, B.; Wang, L.; Qiao, S.; Zeng, X. Effect of antibiotic-

free, low-protein diets with specific amino acid compositions on growth and intestinal flora in weaned pigs. Food Funct. 2020, 11, 493–507.
